# Predicting Waiting Times for Medical Tasks in a Pediatric Hospital Using Machine Learning: Comprehensive, Retrospective, Real-World Study

**DOI:** 10.2196/77297

**Published:** 2025-09-29

**Authors:** Lin Lin Guo, Rui Tang, Jia Yang Wang, Si Zheng, Yin Zeng, Jun Hou, Mo Chen Dong, Jiao Li, Ying Cui

**Affiliations:** 1 Capital Center for Children's Health, Capital Medical University, No.2 Yabao Road, ChaoYang District, Beijing, 100020, China, 86 18380382165; 2 Institute of Medical Information, Chinese Academy of Medical Sciences and Peking Union Medical College, Beijing, China

**Keywords:** waiting time, pediatric hospital, machine learning, queue, medical tasks

## Abstract

**Background:**

The shortage of pediatric medical resources and overcrowding in children’s hospitals are severe issues in China. Accurately predicting waiting times can help optimize hospital operational efficiency.

**Objective:**

This study aims to develop machine learning models to predict waiting times for various laboratory and radiology examinations at a pediatric hospital.

**Methods:**

Time stamp data from laboratory and radiology examinations were retrospectively collected from the pediatric hospital information system between November 1, 2024, and March 13, 2025. Two queue-related and 4 time-based features were extracted using queue theory. Linear regression and 8 machine learning models were trained to predict waiting times for each medical task. Hyperparameters were fine-tuned using randomized search and 10-fold cross-validation, and the bootstrap method was used for model evaluation. Mean absolute error, mean square error, root mean square error, and the coefficient of determination (*R*²) were used as evaluation metrics. Shapley additive explanations values were used to assess feature importance.

**Results:**

A total of 230,864 time-stamped records were included after data preprocessing. The median waiting time was 4.817 (IQR 1.867-12.050) minutes for all medical tasks. Waiting times for radiology examinations were generally longer than those for laboratory tests. Tree-based algorithms, such as random forest and classification and regression trees, performed best in predicting laboratory test waiting times, with *R*² values ranging from mean 0.880(SD 0.003) to mean 0.934 (SD 0.003). However, the machine learning models did not perform well in predicting radiology examination waiting times, with *R*^2^ ranging from 0.114 (SD 0.005) to 0.719 (SD 0.004). Feature importance analysis revealed that queue-related predictors, especially the number of queuing patients, were the most important in predicting waiting times.

**Conclusions:**

Task-specific prediction models are more appropriate for accurately predicting waiting times across various medical tasks. Guided by queue theory principles, we developed machine learning models for the waiting time prediction of each medical task and highlighted the importance of queue-related predictors.

## Introduction

### Background

Pediatric health care resources are considerably more scarce compared to other medical specialties. From 2015 to 2020, the growth rate of categorical pediatric residency positions in the United States was only 7%, which was far slower than the growth rate of other specialties during that time [1]. In China, the lack of resources for pediatric medicine is much more severe. According to national statistical data [2], the number of pediatricians per child in China is approximately half that in the United States. Furthermore, China has a very unequal distribution of pediatric experts, with a heavy concentration in developed metropolitan areas [3]. This leads to patient clustering and increased overcrowding in tertiary pediatric hospitals. A typical pediatric medical visit consists of multiple steps, including consultation, radiology examination or laboratory tests, and medication dispensing. Excessive waiting times at each of these stages not only reduce patient satisfaction [4] but may also contribute to adverse clinical outcomes [5,6]. Therefore, accurately predicting waiting times and improving operational effectiveness in pediatric hospitals is of critical importance.

Queue management systems in hospitals can enhance operational efficiency through features such as real-time queue updates, automated notifications, and online appointment scheduling [7]. Prior research has examined queue-related issues using techniques such as time series analysis (TSA) [8], discrete event simulation [9], and queue theory (QT) [10]. These methods simulate queue dynamics under various parameters to assess operational performance and identify the optimal resource allocation strategies. However, there are several obstacles to overcome before they may be used in actual health care settings. The application of these approaches is limited in practice because health care queues often deviate from the fundamental assumptions that underlie them, such as known initial probability distributions, exponentially distributed waiting times, or stationary queue states [11]. As a result, these models frequently fail to capture the variability and fluctuations inherent in actual queue dynamics, reducing their accuracy in predicting waiting times.

Machine learning models are developed using data-driven approaches and usually do not require restrictive assumptions. To overcome the drawbacks of conventional techniques, machine learning presents a viable substitute for forecasting wait times in hospital lines. In previous studies, Chen et al [12] applied an improved random forest algorithm to predict patient waiting times for each treatment task, such as blood tests, computed tomography (CT) scan, and pharmacy dispensing, and the hospital queuing-recommendation system based on the prediction algorithm reduced waiting times by guiding patient flow. Similarly, Lin et al [13] evaluated the performance of several machine learning algorithms for waiting time prediction and selected the best-performing model for prediction in a pediatric ophthalmology outpatient clinic. Chocron et al [14] compared machine learning algorithms with classical QT models for waiting time prediction and found that machine learning models produced better predictions than traditional methods in complex real-world scenarios. Therefore, machine learning techniques provide a more reliable way to capture variations in actual queue dynamics and improve the prediction accuracy of waiting time. Furthermore, multitask queues in pediatric hospitals have received less attention in the majority of studies, which have concentrated on emergency departments (EDs) [15-17] or radiology departments [18]. Nevertheless, the operational effectiveness of different medical task windows varies, and research focusing on a specific queue may not adequately capture the dynamics of other hospital medical tasks. A comprehensive investigation of waiting time prediction for different medical tasks can offer deeper insights into the use of medical resources and can be used as an alternative method to support medical resource optimization.

### Objectives

This study investigates the dynamic characteristics of multiple medical task queues using real-world data from a large pediatric hospital in northern China. By incorporating principles from QT for feature selection, key features were extracted from actual hospital queue data, and then, we used machine learning techniques to predict waiting times after patient check-in across various medical tasks in the hospital. The predictive performance of different machine learning models is systematically evaluated. Additionally, feature importance analysis is conducted to assess the contribution of individual predictors and to provide insights for optimizing hospital operational efficiency. We also plan to deploy the best-performing model for waiting time prediction to improve the patient experience and help reduce hospital overcrowding in the future.

## Methods

### Problem Definition

During a hospital visit, patients usually have one or more medical tasks, which are frequently spread out over several hospital departments. For instance, a pediatric patient with an acute respiratory tract infection might undergo a series of steps, starting with a consultation in the respiratory department. After blood sampling and throat swab collection, the patient has an X-ray examination and then returns to the pulmonary department when all diagnostic reports are ready and the physician has ordered medicine. Finally, the patient goes to the pharmacy to pick up the necessary medications.

As illustrated, there may be unforeseen waiting times at each step of the medical tasks, which can significantly affect the patient’s overall experience [19]. Furthermore, hospital operational efficiency may be lowered by disorderly queue arrangements. Targeted optimization of medical resource allocation based on key factors influencing waiting time represents a practical and effective strategy. As a result, in actual hospital settings, queue notifications may be provided by predictive algorithms that estimate waiting times with great accuracy, which will assist in reducing patient anxiety.

### Data Collection

This study retrospectively collected records of patients who visited the Capital Center for Children’s Health, Capital Medical University, between November 1, 2024, and March 13, 2025, for various laboratory tests and radiology examinations; the total raw dataset comprised 326,701 entries. The time stamp of these records was retrieved from the health information system for patients who attended the hospital during this period. The key information is summarized in Table 1.

**Table 1.** Information extracted from the health information system.

Records name	Description
Patient ID	A unique identifier assigned to each patient during their visit to the hospital
Medical task name	The specific examination conducted during a patient’s visit, including throat swab, blood sampling, laboratory test for patients with fever, CT^a^, MRI^b^, X-ray, laryngoscopy, ultrasound, and echocardiography
Visit category	The department from which the patient is referred, including outpatient and ED^c^
Service location	The specific location where the patient receives medical services
Check-in time	The time when the patient completes check-in upon arriving for a specific medical task
Sampling or examination start time	The time when the patient begins a specific medical examination: the start time of laboratory tests is recorded as the sampling time, while the start time of the radiology examination is recorded as the examination start time

a CT: computed tomography.

b MRI: magnetic resonance imaging.

c ED: emergency department.

Following the retrieval of all records, the data were arranged according to the practical medical tasks performed by the institution. In this hospital, the tasks for laboratory tests include throat swab at the first floor (throat swab–first floor) and the second floor (throat swab–second floor), blood sampling, and laboratory test for patients with fever (laboratory test–fever). The radiology examination includes CT, magnetic resonance imaging (MRI), X-ray, laryngoscopy, ultrasound, and echocardiography. Blood sampling, ultrasound, and echocardiography are further divided into outpatient and ED windows at this facility.

### Ethical Considerations

All data used in our study was anonymized and deidentified and did not involve data related to humans. Therefore, our research was exempted from the requirement of written informed consent and was approved by the Ethics Committee of the Capital Center for Children's Health, Capital Medical University (SHERLLM2024037).

### Data Preprocessing

We define the waiting time as the duration from the patient’s check-in to the start of the sampling or examination, as follows:


(1) Waiting time=Sampling/Examination start time−Check-in time

First, we removed records that do not include the sampling or examination start time and check-in time. Due to the presence of numerous duplicate records in the health information system, we then performed a deduplication procedure on the extracted data. If the patient ID and sampling or examination start time are identical in the records, it will be removed as a redundant duplicate. To avoid negative waiting times, we then removed data when the check-in time is later than the sampling or examination start time. In addition, there may be very long waiting time outliers because some patients may not line up for the medical service window immediately after check-in—possibly leaving the hospital for a period or coming back on a subsequent day to continue their tests or examinations. To address this issue, we applied an IQR method to denoise the data using the following criterion:

Noise record§gt;75% quantile+1.5×(75% quantile−25% quantile) (2)

Records with waiting times exceeding the IQR were classified as noise and discarded. Model development was then conducted using the remaining data.

### Feature Construction

Queue-length (QL) predictors and delay-history predictors are the 2 types of predictors used in earlier QT experiments to forecast waiting times [20]. While the latter makes predictions based on past waiting times, the former refers to projecting waiting time based on the queue’s present status factors. As we have access to the precise time stamps for every patient’s test or examination, we make predictions using QL predictors, which comprise the following two categories of features:

1. Time-based feature; these include the month, day, hour, and day of the week when a patient undergoes a specific medical task. These indicators can all be derived from the abovementioned records. Given that patient data vary significantly across different periods, these 4 time-related factors were considered as potential predictors.
2. Queue-related feature; these include the patient arrival rate per hour, calculated by counting the number of patients registering for a particular task each hour, and the number of queuing patients, that is, the number of patients who have checked in but have not yet started the sampling or examination.

In summary, we selected only 6 indicators, including month, day, hour, day of the week, arrival rate, and the number of queuing patients, to predict the waiting time using a concise set of predictors.

### Model Development and Feature Importance Analysis

In our experiment, we use linear regression (LR) as a baseline model and 8 machine learning models for waiting time prediction, including k-nearest neighbor (KNN), support vector regression (SVR), classification and regression tree (CART), elasticNet, random forest (RF), light gradient boosting machine (LightGBM), extreme gradient boosting (XGBoost), and multilayer perceptron (MLP). As the waiting time is a continuous variable, we evaluate the models using the following performance metrics: mean absolute error (MAE), mean square error (MSE), root mean square error (RMSE), and the coefficient of determination (*R*²). Equations 3–6 show the formula of MAE, MSE, RMSE, and *R*^2^, where the ${\hat{y}}_{i}$ is the prediction value, the ${\overset{-}{y}}_{i}$ represents the mean value of the waiting time, and the $y_{i}$ is the actual value.


(3) $$MAE=\sum_{i=1}^{n}\frac{|{\hat{y}}_{i}-y_{i}|}{n}$$

(4) $$MSE=\sum_{i=1}^{n}\frac{({\hat{y}}_{i}-y_{i}{)}^{2}}{n}$$

(5) $$RMSE=\sqrt{\sum_{i=1}^{n}\frac{({\hat{y}}_{i}-y_{i}{)}^{2}}{n}}$$

(6) $$R^{2}=1-\frac{\sum_{i=1}^{n}({\hat{y}}_{i}-y_{i}{)}^{2}}{\sum_{i=1}^{n}(y_{i}-\bar{y}{)}^{2}}$$

**Figure 1.**
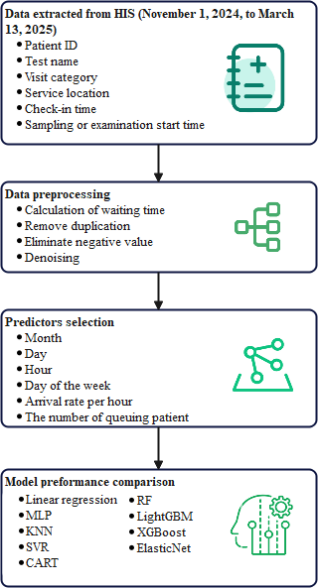
Flowchart of waiting time prediction. CART: classification and regression tree; HIS: health information system; KNN: k-nearest neighbor; LightGBM: light gradient boosting machine; MLP: multilayer perceptron; RF: random forest; SVR: support vector regression; XGBoost: extreme gradient boosting.

A randomized search and 10-fold cross-validation were used to fine-tune each model’s hyperparameters. The performance of the models was evaluated using the abovementioned metrics, and the model with the best performance across these metrics was selected for prediction. To further evaluate the stability and reliability of the chosen model, a bootstrap approach was applied. MAE, MSE, RMSE, and *R*^2^ were calculated for each bootstrap sample by comparing the model’s predictions with the true values. The 95% CIs for each metric were derived to quantify the uncertainty in the model’s predictive performance. Feature contributions to the waiting time prediction were quantified using Shapley additive explanations (SHAP) values [21]. For tree-based models, SHAP values were computed directly; for others (KNN and SVR), a background dataset was used to simulate feature absence, allowing for marginal contribution estimation via the KernelExplainer function. The entire research process is shown in Figure 1. All analyses were performed using Python (version 3.12; Python Software Foundation).

## Results

### Basic Characteristics of the Medical Queue

From November 1, 2024, to March 13, 2025, a total of 230,864 records were included after preprocessing the raw data. The required tasks were classified into 2 main categories: laboratory tests, which included 5 types of tasks, and radiology examinations, which included 8 types of tasks. The most frequently performed laboratory test was blood sampling for outpatient patients (76,587/230,864, 33.17%), while the most common radiology examination was X-ray (31,125/230,864, 13.48%; Table 2).

**Table 2.** Basic characteristics of queuing in different medical tasks (N=230,864).

Tests and examinations		Tasks, n (%)	Time, range (min)	Time, median (IQR)
Laboratory test				
	Throat swab—first floor	26,314 (11.05)	0.017‐28.4	1.617 (0.483‐5.799)
	Throat swab—second floor	13,296 (5.75)	0.017‐26.967	1.467 (0.417‐6.971)
	Blood sampling—ED^a^	23,558 (10.20)	0.150‐5.533	1.650 (1.049‐2.483)
	Blood sampling—outpatient	76,587 (33.17)	0.199‐25.5	3.225 (2.583‐10.700)
	Laboratory test—fever	9533 (4.13)	0.017‐8.667	1.783 (1.017‐3.349)
Radiology examination				
	Ultrasound—ED	22,601 (9.79)	0.199‐48.050	10.883 (5.283‐20.717)
	Ultrasound—outpatient	8031 (3.48)	0.517‐85.299	23.400 (12.750‐37.067)
	Echocardiography—ED	394 (0.17)	0.033‐9.683	3.958 (3.054‐5.212)
	Echocardiography—outpatient	5067 (2.19)	0.550‐7.983	3.200 (2.600‐4.167)
	Laryngoscope	4856 (2.10)	0.467‐43.267	11.975 (5.217‐19.771)
	X-ray	31125 (13.48)	0.065‐6.566	5.106 (10.872‐21.852)
	MRI^b^	3324 (1.44)	0.008‐80.157	8.389 (17.825‐33.262)
	СТ^c^	6178 (2.68)	0.002‐19.412	2.406 (3.933‐6.947)

a ED: emergency department.

b MRI: magnetic resonance imaging.

c CT: computed tomography.

**Figure 2.**
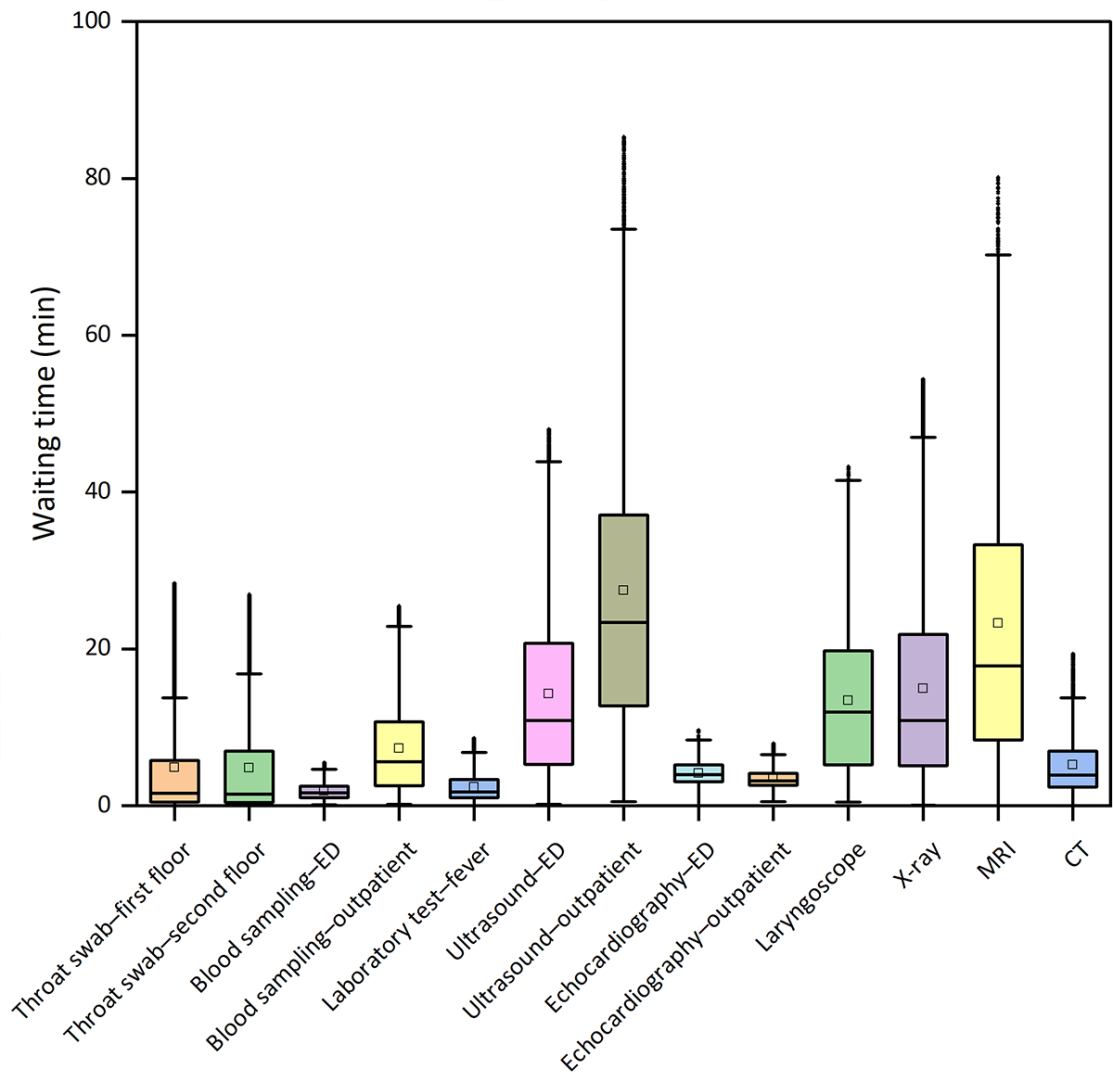
Box plot of distribution of waiting time. CT: computed tomography; ED: emergency department; MRI: magnetic resonance imaging.

In the laboratory test category, we observed that blood sampling–ED (median 1.650, IQR 1.049-2.483 min) had the narrowest waiting time range, while the task for blood sampling–outpatient (median 3.225, IQR 2.583-10.700 min) had a relatively extensive waiting time range (Figure 2). For radiology examinations, the range of waiting time for the echocardiography task was narrow (median 3.958, IQR 3.054-5.212 min for ED; median 3.200, IQR 2.600-4.167 min for outpatient). In contrast, outpatients had to wait much longer for an ultrasound (median 37.067, IQR 12.750-23.400 min; Figure 2). Compared to laboratory tests, patients experienced longer waiting times in most radiology examinations.

The distribution of waiting time of the total medical task followed an exponential pattern (Figure S1 in Multimedia Appendix 1). At different time points, we observed average arrival rate peaks for medical tasks at approximately 10:00 AM, 15:00 PM, and 8:00 PM. Similarly, 3 peaks were also present for the average number of queuing patients across different days of the week (Figures S2 and S3 in Multimedia Appendix 1). However, the waiting time of patients showed a first earlier peak at approximately 7:00 AM, with 2 lower peaks at approximately 12:00 AM and 8:00 PM (Figure S4 in Multimedia Appendix 1). In addition, the distribution of waiting time for different medical tasks before and after denoising is shown in Figure S5 in Multimedia Appendix 1.

### Model Evaluation

**Figure 3.**
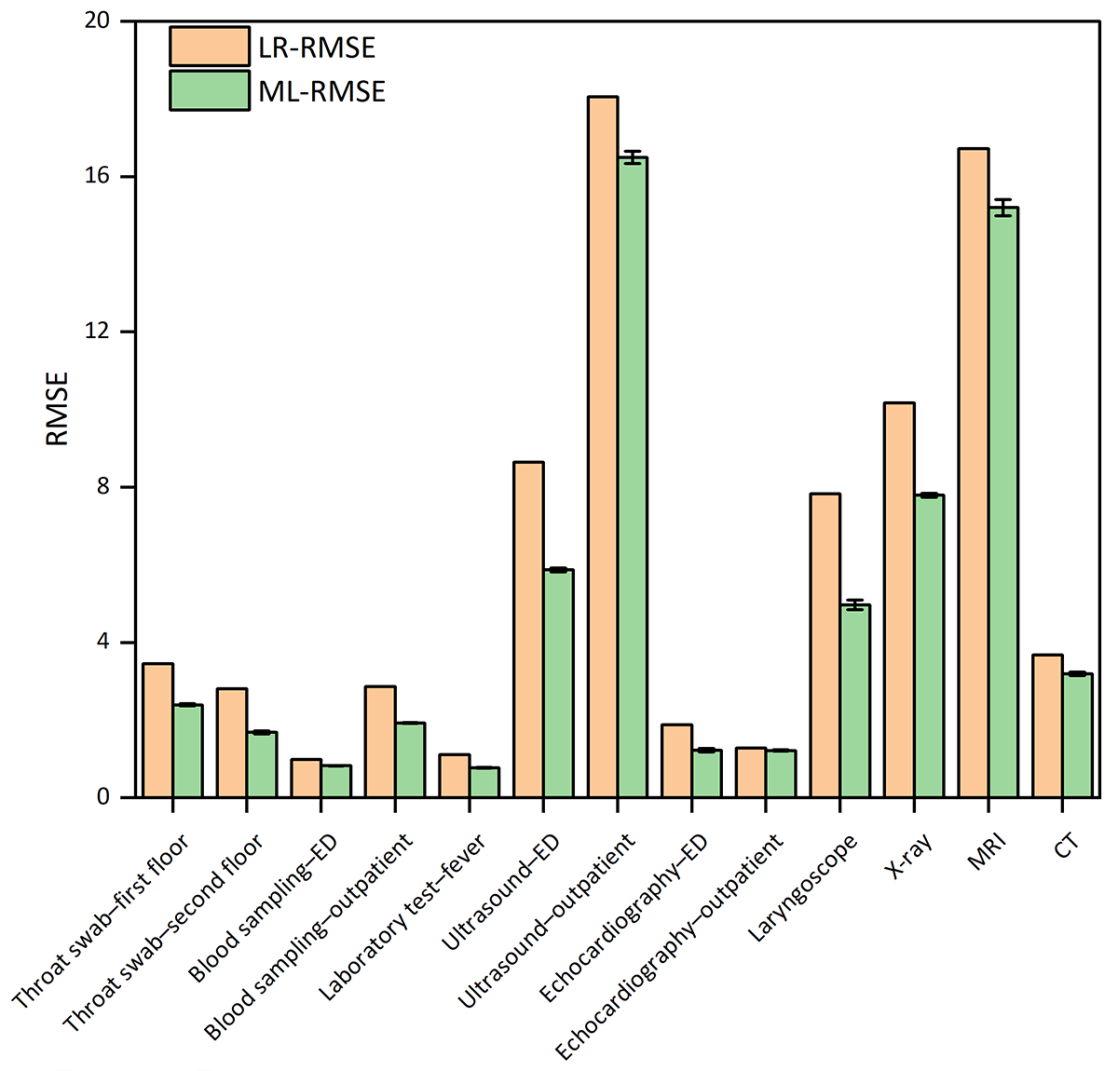
Root mean square error (RMSE) of different machine learning (ML) models for each medical task compared with linear regression (LR). CT: computed tomography; ED: emergency department; MRI: magnetic resonance imaging.

We used 8 different models for predicting waiting times before laboratory and radiology tasks, while LR was used as the baseline model. After hyperparameter optimization, the optimal performance of the selected models for different tasks is shown in Table S1 in Multimedia Appendix 1. In 6 medical task queues, RF demonstrated the best predictive performance, with RMSE ranging from 1.925 (SD 0.015) to 2.395 (SD 0.033) for laboratory tests and from 1.217 (SD 0.015) to 15.204 (SD 0.207) for radiology examinations. In addition, XGBoost demonstrated effective performance in predicting waiting times for the throat swab–first floor (RMSE: mean 2.395, SD 0.033; *R*^2^: mean 0.880, SD 0.003), echocardiography-ED (RMSE: mean 1.224, SD 0.048; *R*^2^: mean 0.612, SD 0.021), and CT (RMSE: mean 3.191, SD 0.043; *R*^2^: mean 0.382, SD 0.011), and CART, LightGBM, SVR, and KNN can also be used for waiting time prediction. All selected models outperformed the baseline LR model (Figures3  4). The optimal hyperparameters identified through randomized search are provided in Table S2 in Multimedia Appendix 1. In addition, the calibration and residual plots are provided in Figures S8-S18 in Multimedia Appendix 1.

**Figure 4.**
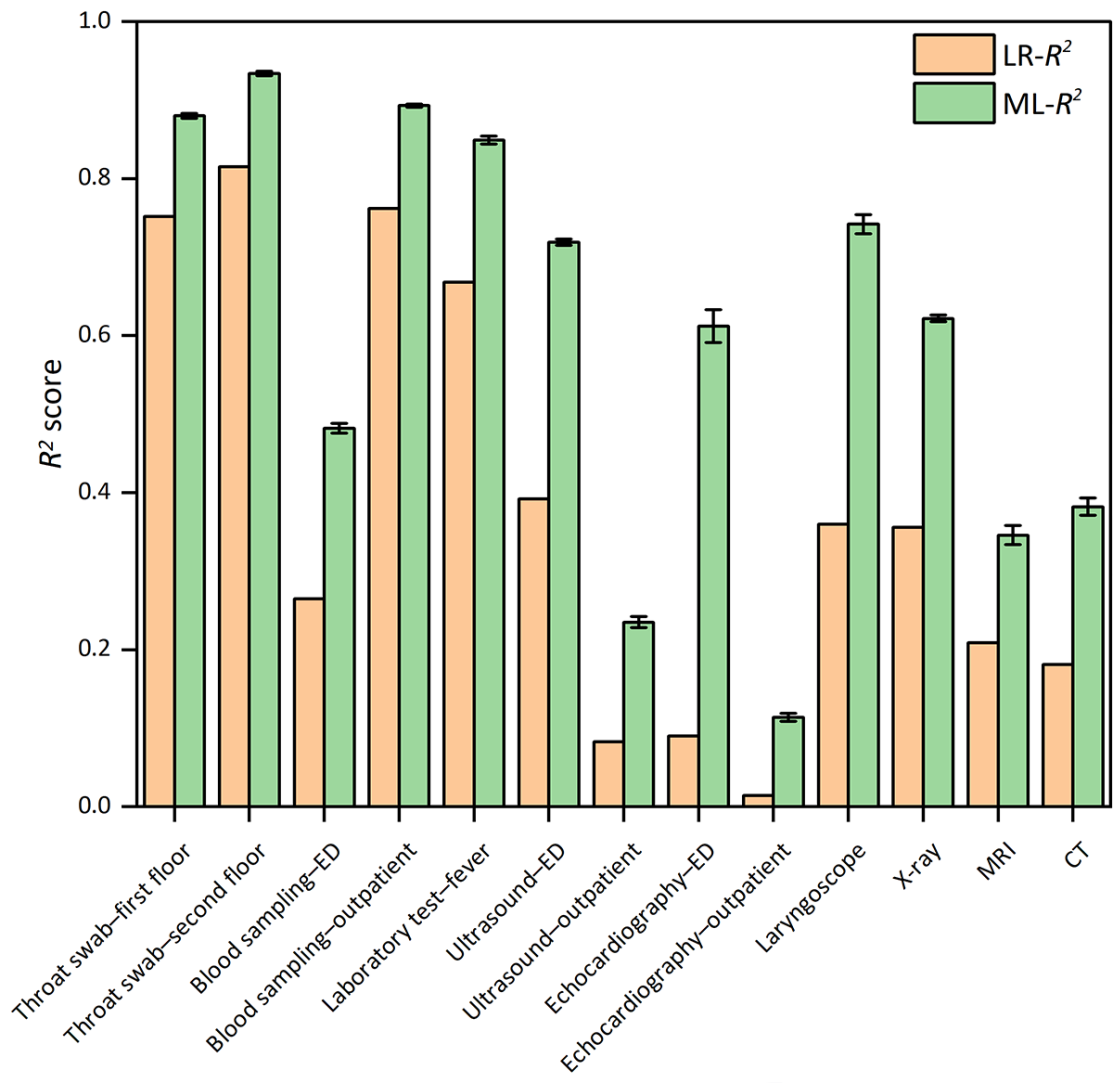
*R*2 score of different machine learning (ML) models for each medical task compared with linear regression (LR). CT: computed tomography; ED: emergency department; MRI: magnetic resonance imaging.

### Feature Importance Analysis

**Figure 5.**
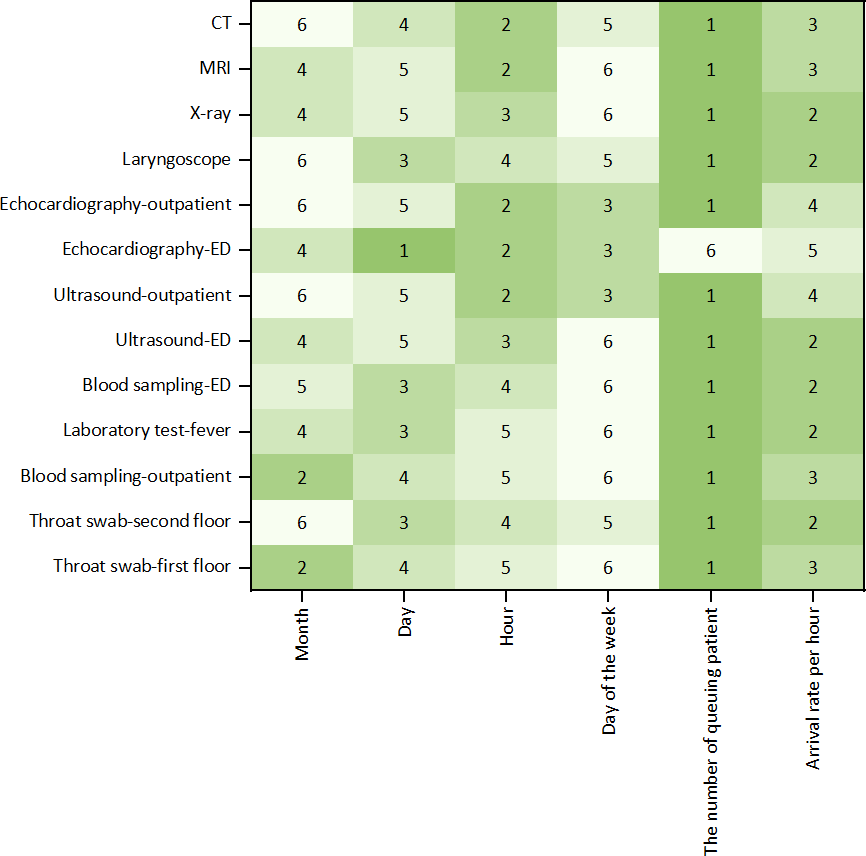
Heat map of feature importance ranking in different models (x-axis: predictive features; y-axis: medical tasks). The numbers in each cell represent the ranking order obtained based on the Shapley additive explanations values. CT: computed tomography; ED: emergency department; MRI: magnetic resonance imaging.

On the basis of the QT principle, we ultimately selected the number of queuing patients, arrival rate, month, day, day of the week, and hour as independent features for predicting waiting time. To evaluate the contribution of each feature to the machine learning model, we computed SHAP values, which quantify the impact of individual features on the predicted waiting time. The mean SHAP value was used to rank each feature. The rankings of feature importance across different models are visualized in a heat map plot (Figure 5). Notably, for the majority of medical tasks, queue-related features, such as the number of queuing patients, emerged as the most influential predictor of waiting time, and the arrival rate was also expressed as another important feature for waiting time prediction. The details of the mean SHAP value are provided in Table S3 in Multimedia Appendix 1.

## Discussion

### Principal Findings

This study collected patient data from the Capital Center for Children’s Health, Capital Medical University, spanning the period from November 1, 2024, to March 13, 2025, comprising a total of 230,864 records after data preprocessing. The distribution of waiting times for overall medical tasks followed an exponential pattern, with a median value of 4.817 minutes. Notably, peak periods in the average patient arrival rate and the average number of queuing patients were observed at 10:00 AM, 3:00 PM, and 8:00 PM. The waiting time exhibited a different pattern, with an earlier peak occurring at approximately 7:00 AM, along with 2 additional, smaller peaks around 12:00 PM and 8:00 PM, indicating the periods of highest congestion within the hospital. During model selection, tree-based algorithms such as RF, XGBoost, CART, and LightGBM demonstrated better accuracy in predicting waiting times for most medical task queues, while SVR and KNN can also be used as appropriate algorithms. Additionally, feature importance analysis revealed that the number of queuing patients was the most influential predictor of waiting time.

QT and TSA are classical approaches used to model and simulate queue dynamics in health care settings [22,23]. The application of QT requires the key input parameters, including the average arrival rate (λ) and average service rate (μ). Several queue parameters, including average waiting time (Wq), total time spent in the system (Ws), average QL (Ls), and average number of individuals waiting (Lq), can be calculated based on these inputs. For example, waiting time can be estimated using the formula $W_{q}=\frac{\lambda }{\mu (\mu -\lambda )}$ in an M/M/1 queue [24]. By simulating the queuing status and adjusting service rates, different operational scenarios can be modeled for service optimization. However, QT has several limitations when applied to the prediction of waiting times in real-world hospital settings. First, as demonstrated in our study, QT assumes that patient arrivals follow a Poisson process and that service times conform to exponential distributions. While the entire waiting time across all tasks approximates an exponential distribution in our study, this assumption does not universally hold for each medical task queue (the distribution of waiting times for each task was provided on our Github website). Second, QT estimates queue parameters relying on average arrival and service rates, which are dynamic in clinical practice. Because patient volume and service effectiveness are constantly changing, QT’s static assumptions are inadequate for simulating operating situations in real time [25]. Third, accurately measuring the actual service rates for each medical task is challenging. As a result, we had to rely on estimations based on prior experience, which likely reduced the precision of the modeled parameters. Additionally, TSA, which involves modeling sequences of observations indexed over time, has been applied in previous studies to forecast ED attendances [8,23,26-28]. While effective at capturing periodic trends in patient volumes, TSA is more suited to predicting daily volumes rather than individual-level waiting times. Instead of depending on the strict presumptions of QT or TSA, machine learning models may directly use real-world waiting time data, enabling flexible, data-driven prediction.

This study assessed a variety of models for the waiting time prediction task to achieve the optimal predictive performance. In most scenarios, the tree-based model performed better than other models and demonstrated strong predictive capability. The RMSE of the models in our study was lower than that reported in previous studies [11,12,17,29]. Strong predictive performance was shown by high *R*^2^ values for a number of tasks, including throat swab–second floor (*R*²: mean 0.934, SD 0.003), blood sampling–outpatient (*R*²: mean 0.893, SD 0.002), and throat swab–first floor (*R*²: mean 0.880, SD 0.003). As widely recognized, machine learning models are generally more effective than LR in capturing nonlinear relationships [30], and tree-based models typically handle tabular data more efficiently than deep learning models [31]. However, for several tasks, such as MRI, CT, ultrasound-outpatient, and echocardiography-outpatient queues, we observed weak associations between the features and waiting time. We believe that the small sample sizes for these tasks may contribute to the poor model performance. A longer data collection period could help improve performance. Additionally, the 6 features used in our model may not be sufficient for predicting waiting times across all medical tasks. Some medical tasks may have inherent complexities or variability in waiting times that are not fully captured by the current available features. For example, the waiting time for outpatient ultrasound and MRI may depend on factors such as the age and urgency of the case or physician workload, which are difficult to quantify with the current feature set. While we aim to develop a lightweight prediction model with simplified features for easier deployment, it is clear that for certain tasks, feature engineering and the inclusion of additional variables—such as patient age, gender, department, and diagnosed diseases—are necessary to improve performance. These strategies can help improve the prediction models for underperforming tasks and support their future deployment.

Notably, the data used to train our predictive models were primarily derived from the first and fourth quarters of the year, when patient visit patterns are different from those observed in other periods. According to surveillance data from the Chinese Center for Disease Control and Prevention, the prevalence of acute respiratory infections is elevated between November 2024 and January 2025 [32-34]. This seasonal trend aligns with epidemiological surveillance data from the United States, which indicate a predominance of respiratory tract infections among children during the winter months [35,36]. Throughout this time, pediatric attendance was consistently high from late December to January (Figure S19 in Multimedia Appendix 1), with January having the most visits (44,808/145,739, 30.75%). This phenomenon is probably caused by a confluence of environmental and school-related factors: lower temperatures and humidity promote viral stability and propagation, while more interpersonal interaction in school facilitates the spread of infectious illnesses. Children’s hospital visits are accelerated by these 2 factors. The most frequent combinations for pediatric patients who received multiple medical services during a single visit were related to acute respiratory infections, including throat swab → laryngoscopy → X-ray → echocardiography, throat swab → X-ray → blood sampling, and throat swab → blood sampling [37,38]. These findings imply that queue loads for various services are considerably impacted by fluctuations in illness occurrence. Although feature importance analysis suggests that the contribution of the month feature is not particularly important across different prediction models, this may be attributed to the short data collection period, which constrains the ability to capture long-term temporal trends. Therefore, seasonal demand fluctuations can also be considered when optimizing patient flow and resource allocation (Figure S20 in Multimedia Appendix 1). Strategies such as increasing the number of service windows during high-demand periods could alleviate queue burdens and improve operational efficiency. For example, at times when respiratory infections were most common at this institution, a second throat swab window was opened.

### Limitations

This study has several limitations. First, our study is a single-center investigation that was mostly carried out at a pediatric hospital during the winter. The findings of this study might not apply to circumstances outside of a children’s hospital due to the unique characteristics of the patients at this facility. Second, although medicine dispensing is a critical component of the clinical workflow, it was excluded from the analysis since the pharmacy did not have a procedure for patients to check in, making it impossible to calculate waiting times. Lastly, waiting times for various medical tasks were predicted using numerous models, which complicated the overall predictive framework and posed challenges for real-world implementation.

### Conclusions

In conclusion, the distribution of patient waiting times exhibits 3 distinct peak periods. However, waiting time patterns differ markedly across various medical task queues, each displaying unique characteristics that do not align with the overall trend. Consequently, developing task-specific predictive models for each medical task queue can enhance prediction accuracy. Feature importance analysis reveals that although queue-related features are the most influential in predicting patient waiting time, time-based features might also contribute meaningfully and should be considered to further optimize hospital operational efficiency.

## Supplementary material

10.2196/77297 Multimedia Appendix 1 Distribution of waiting time, hyperparameters, and performance of the model.

## Abbreviations

CART: classification and regression tree
CT: computed tomography
ED: emergency department
KNN: k-nearest neighbor
LightGBM: light gradient boosting machine
LR: linear regression
MAE: mean absolute error
MLP: multilayer perceptron
MRI: magnetic resonance imaging
MSE: mean square error
QL: queue length
QT: queue theory
RF: random forest
RMSE: root mean square error
SHAP: Shapley additive explanations
SVR: support vector regression
TSA: time series analysis
XGBoost: extreme gradient boosting
