# Supplementary material for: Predicting Waiting Times for Medical Tasks in a Pediatric Hospital Using Machine Learning: Comprehensive, Retrospective, Real-World Study
Source: JMIR Med Inform. 2025 Sep 29;13:e77297. doi: 10.2196/77297 (PMC12481172; doi:10.2196/77297)
Supplement: Multimedia Appendix 1 [file medinform-v13-e77297-s001.docx]

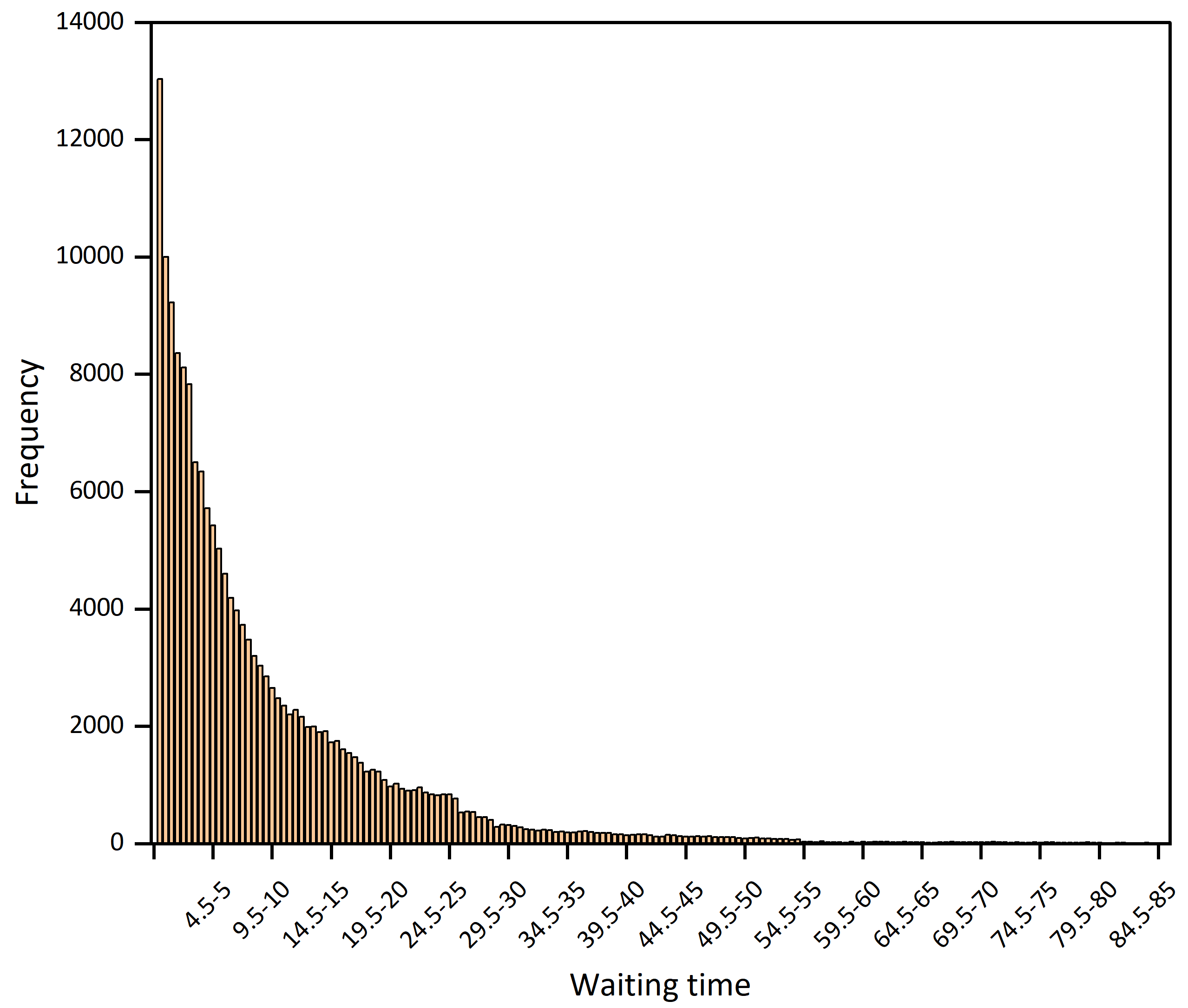


**Figure S1.** Distribution of waiting time across all medical tasks


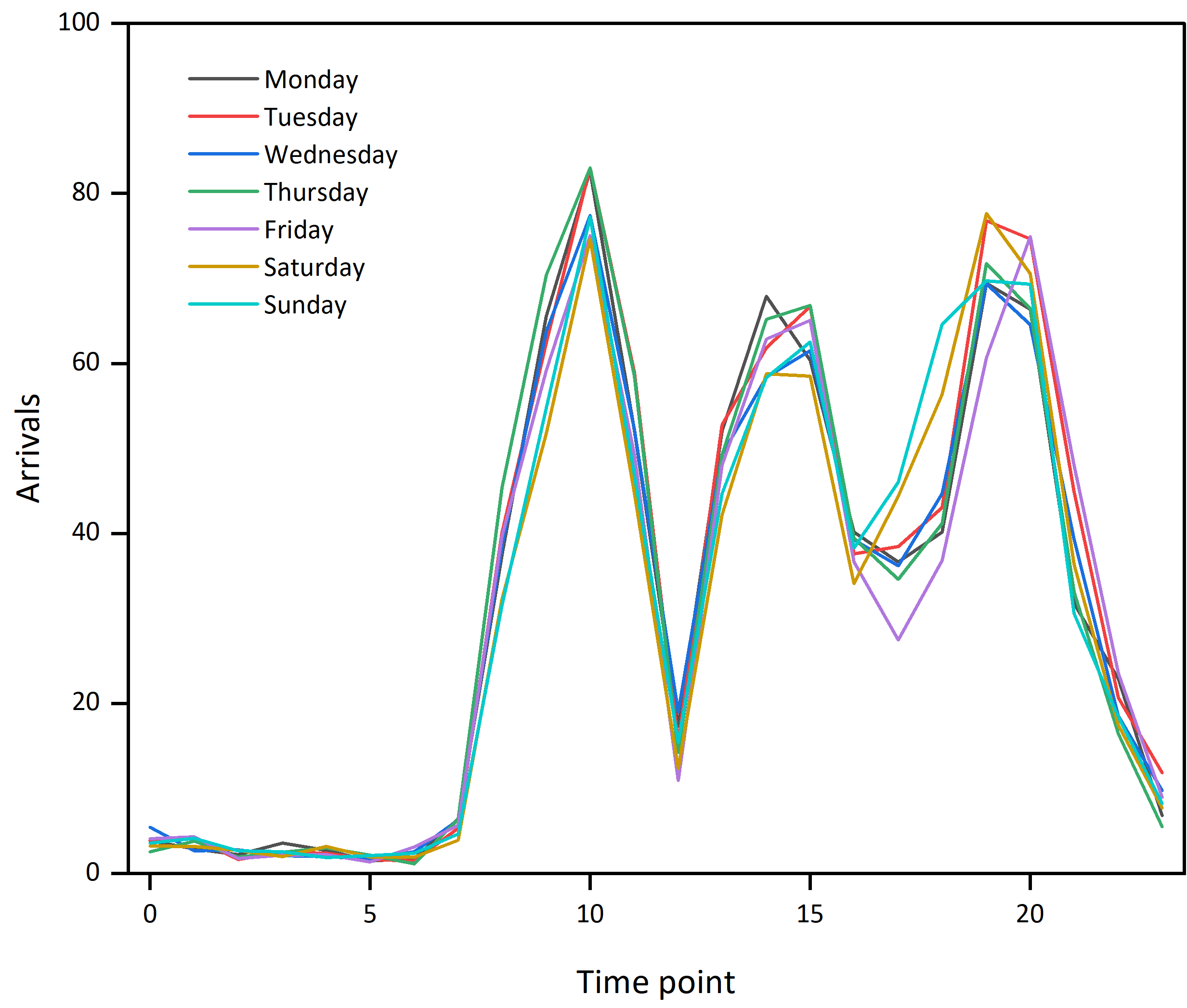


**Figure S2.** Average arrival rate as a function of time of day and day of week


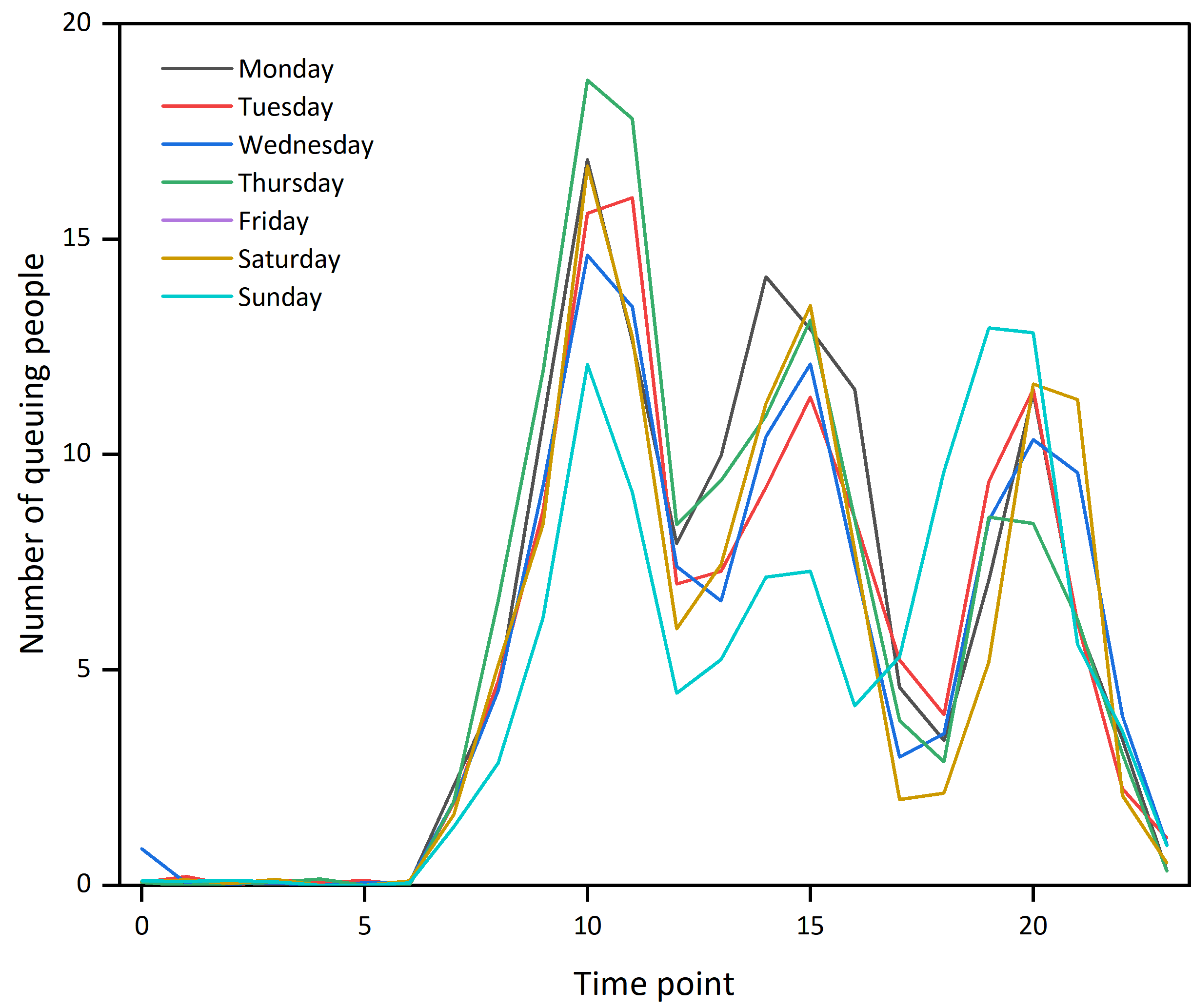


**Figure S3.** Average number of queuing people as a function of time of day and day of week


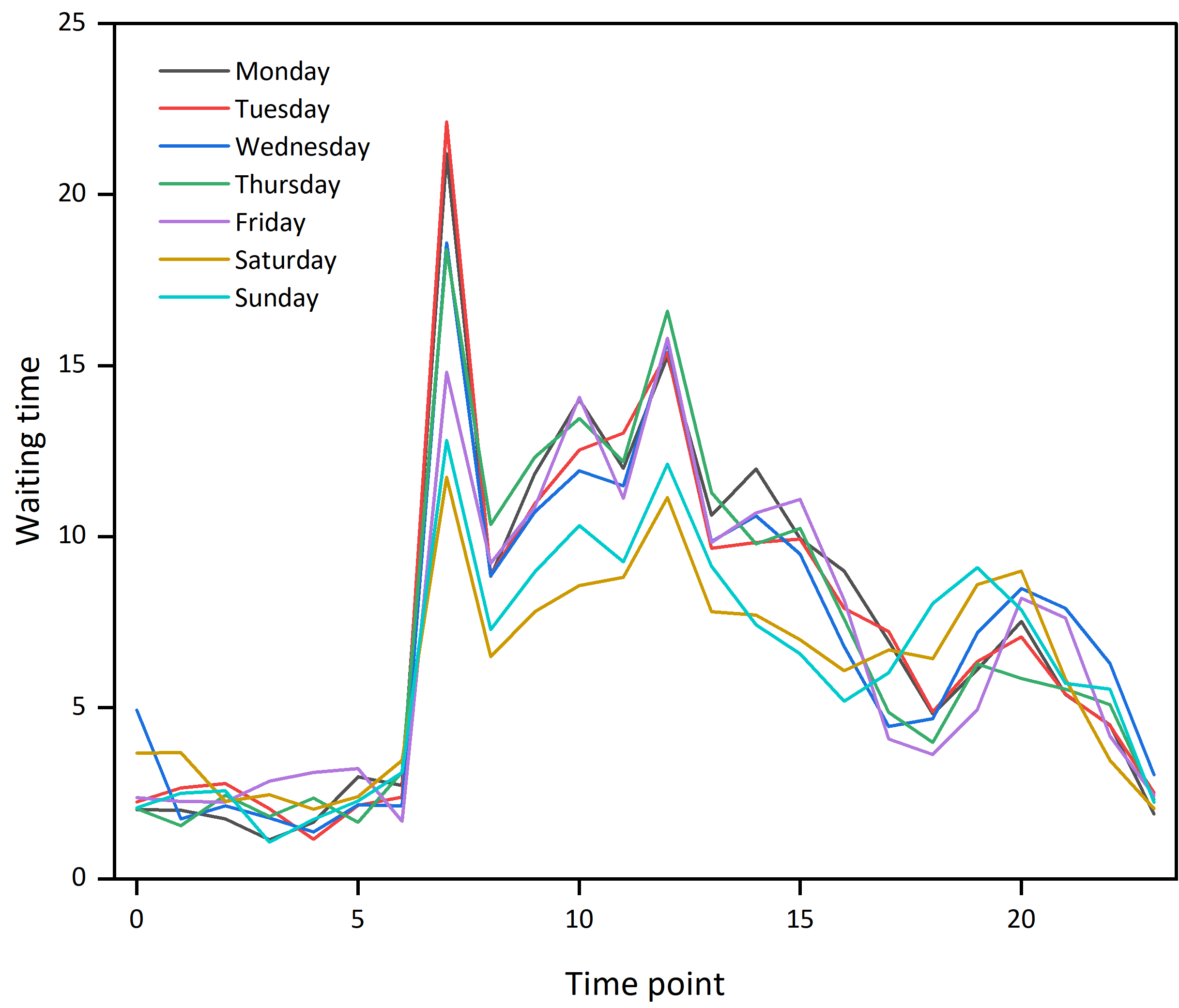


**Figure S4.** Average waiting time as a function of time of day and day of week


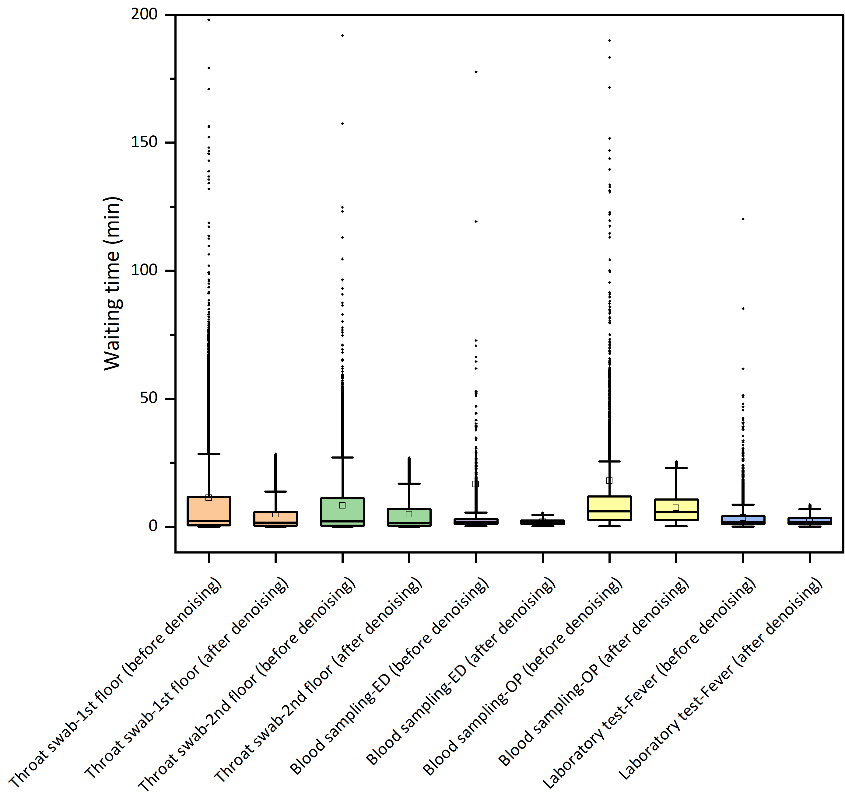


**(A)** Laboratory test


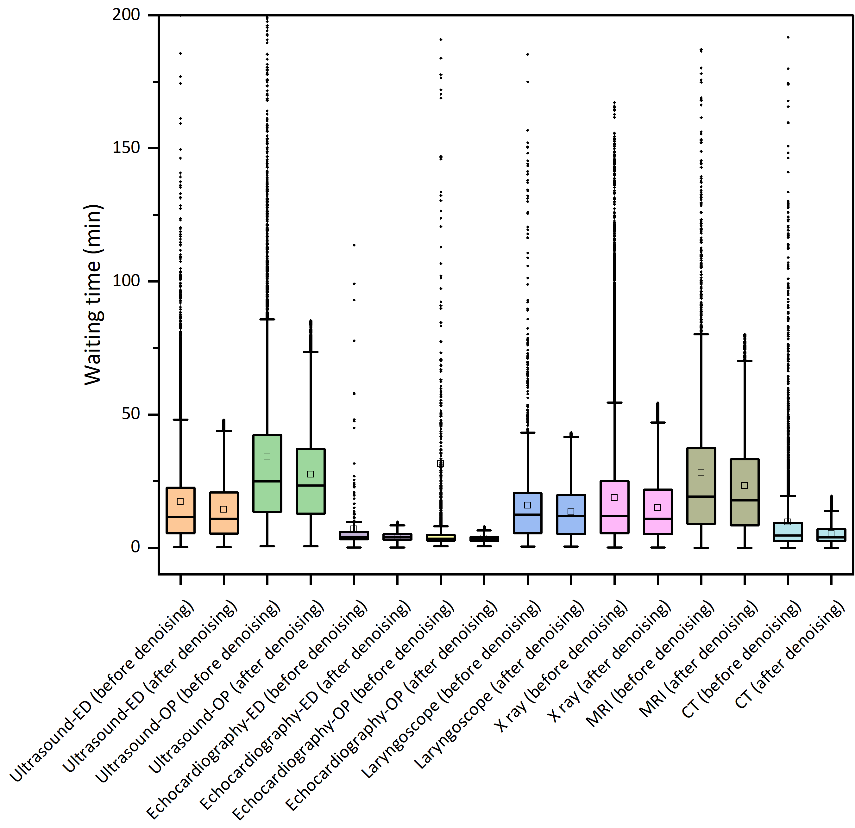


**(B)** Radiology examination

**Figure S5.** Distribution of waiting time for different medical tasks before and after denoising

**Note:** only the range from 0 to 200 is shown.

**Table S1.** Performance of the best machine learning model for waiting time prediction of each medical task

|  | **Model** | **Mean ± 95% Confidence Interval** | | | |
| --- | --- | --- | --- | --- | --- |
|  |  | **MAE** | **MSE** | **RMSE** | **R^2^** |
| **Laboratory test** | | | | | |
| Throat swab-1^st^ floor | RF | 1.180 ± 0.013 | 5.736 ± 0.159 | 2.395 ± 0.033 | 0.880 ± 0.003 |
| Throat swab-2^nd^ floor | CART | 0.894±0.012 | 2.833±0.127 | 1.683±0.038 | 0.934±0.003 |
| Blood sampling-ED | SVR | 0.628 ± 0.004 | 0.683 ± 0.009 | 0.827 ± 0.005 | 0.482 ± 0.006 |
| Blood sampling-OP | RF | 1.243±0.005 | 3.705±0.057 | 1.925±0.015 | 0.893±0.002 |
| Laboratory test-Fever | KNN | 0.513 ± 0.006 | 0.593 ± 0.019 | 0.770 ± 0.012 | 0.849 ± 0.005 |
| **Radiology examination** | | | | | |
| Ultrasound-ED | RF | 3.873 ± 0.030 | 34.447 ± 0.590 | 5.869 ± 0.050 | 0.719 ± 0.004 |
| Ultrasound-OP | LightGBM | 12.451 ± 0.115 | 272.049 ± 5.272 | 16.493 ± 0.160 | 0.235 ± 0.007 |
| Echocardiography-ED | XGBoost | 0.943 ± 0.038 | 1.501 ± 0.118 | 1.224 ± 0.048 | 0.612 ± 0.021 |
| Echocardiography-OP | RF | 0.941 ± 0.011 | 1.482 ± 0.038 | 1.217 ± 0.015 | 0.114 ± 0.005 |
| Laryngoscope | SVR | 2.843 ± 0.060 | 24.730 ± 1.222 | 4.971 ± 0.123 | 0.742 ± 0.012 |
| X ray | RF | 5.229±0.033 | 60.722±0.718 | 7.792±0.046 | 0.622±0.004 |
| MRI | RF | 12.013 ± 0.158 | 231.205 ± 6.297 | 15.204 ± 0.207 | 0.346 ± 0.012 |
| CT | XGBoost | 2.306 ± 0.028 | 10.184 ± 0.275 | 3.191 ± 0.043 | 0.382 ± 0.011 |

**Table S2.** Hyperparameter in the final model

| **Prediction model** | **Hyperparameter** |
| --- | --- |
| Throat swab-1st floor-RF | 'n_estimators': 357,  'max_depth': 14,  'min_samples_leaf': 1,  'max_features': 0.4021767833180502,  'bootstrap': True |
| Throat swab-2nd floor-CART | 'max_depth': 18,  'min_samples_split': 6,  'min_samples_leaf': 3,  'max_features': 0.6405074202323546 |
| Blood sampling-ED-SVR | 'C': 2.8110563835485087,  'epsilon': 0.5359881736867094,  'gamma': 'auto' |
| Blood sampling-OP-RF | 'n_estimators': 441,  'max_depth': 26,  'min_samples_leaf': 3,  'max_features': 0.3984879476706409,  'bootstrap': True |
| Laboratory test-Fever-KNN | 'n_neighbors': 10,  'weights': 'distance',  'p': 2 |
| Ultrasound-ED-RF | 'n_estimators': 279,  'max_depth': 39,  'min_samples_leaf': 4,  'max_features': 0.4079929080384635,  'bootstrap': True |
| Ultrasound-OP-LightGBM | 'max_depth': 10,  'num_leaves': 32,  'learning_rate': 0.05376724985872417,  'feature_fraction': 0.7062344006015898,  'bagging_fraction': 0.5579990604600252 |
| Echocardiography-ED-XGBoost | 'n_estimators': 267,  'max_depth': 12,  'learning_rate': 0.012034582723863858,  'subsample': 0.927244701816625,  'colsample_bytree': 0.6556575947392053,  'gamma': 0.4881915805341791,  'min_child_weight': 3,  'reg_alpha': 0.17975496372783134,  'reg_lambda': 0.532576559712103 |
| Echocardiography-OP-RF | 'n_estimators': 106,  'max_depth': 10,  'min_samples_leaf': 4,  'max_features': 0.31154400313415803,  'bootstrap': False |
| Laryngoscope-SVR | 'C': 13.374107709675418,  'epsilon': 0.9837057502857758,  'gamma': 'auto' |
| X ray-RF | 'n_estimators': 167,  'max_depth': 15,  'min_samples_leaf': 4,  'max_features': 0.43521568067567795,  'bootstrap': False |
| MRI-RF | 'n_estimators': 434,  'max_depth': 29,  'min_samples_leaf': 5,  'max_features': 0.3001632304422872,  'bootstrap': False |
| CT-XGBoost | 'n_estimators': 355,  'max_depth': 6,  'learning_rate': 0.01853665883041131,  'subsample': 0.8450569000800362,  'colsample_bytree': 0.7550732724828378,  'gamma': 0.2288611748518899,  'min_child_weight': 8,  'reg_alpha': 0.28331316993642636,  'reg_lambda': 0.670456025846887 |


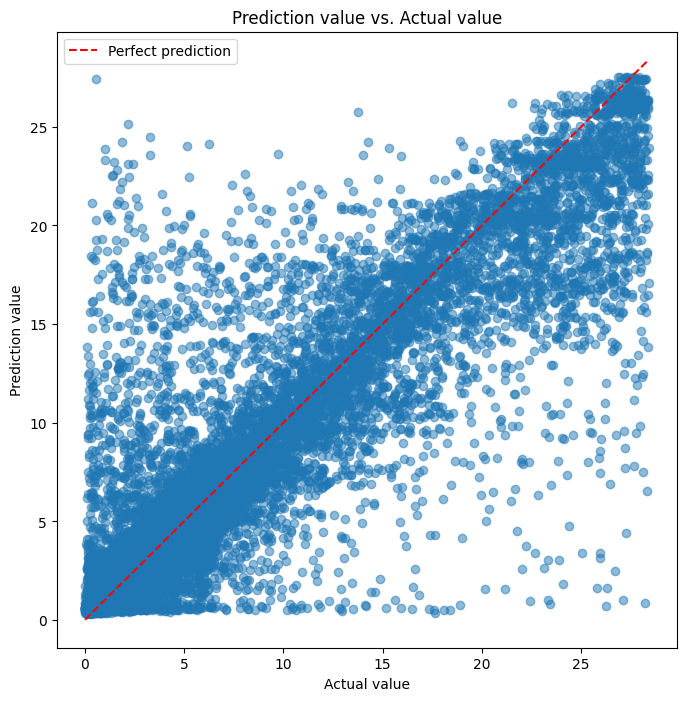

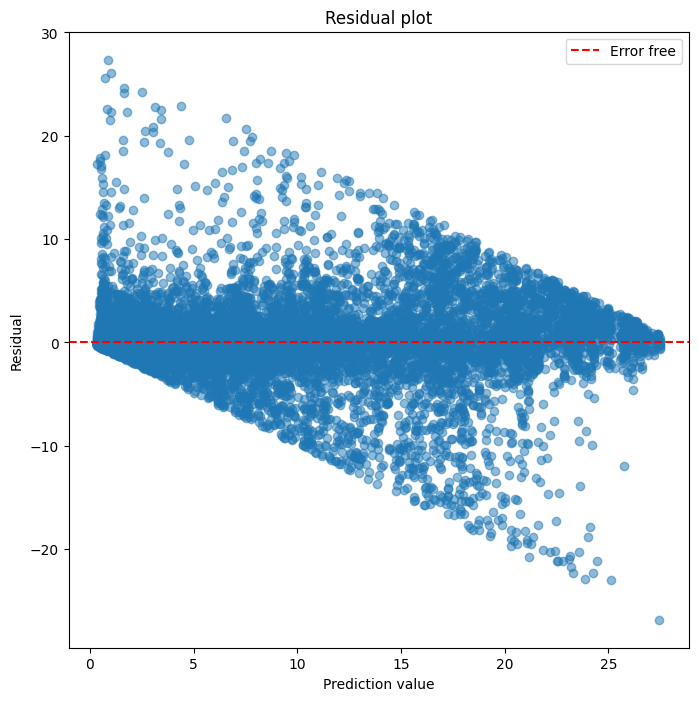


(A) Calibration plot (B) Residual plot

**Figure S6** Throat swab-1st floor


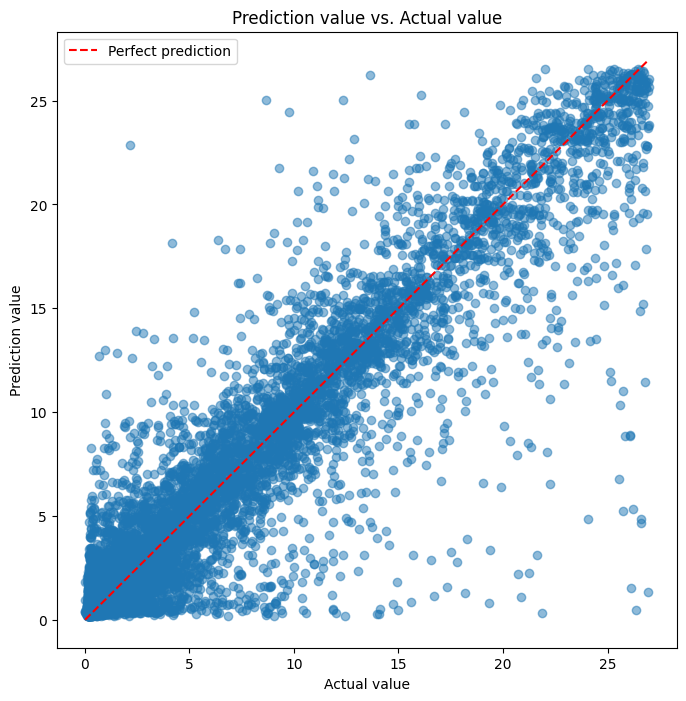

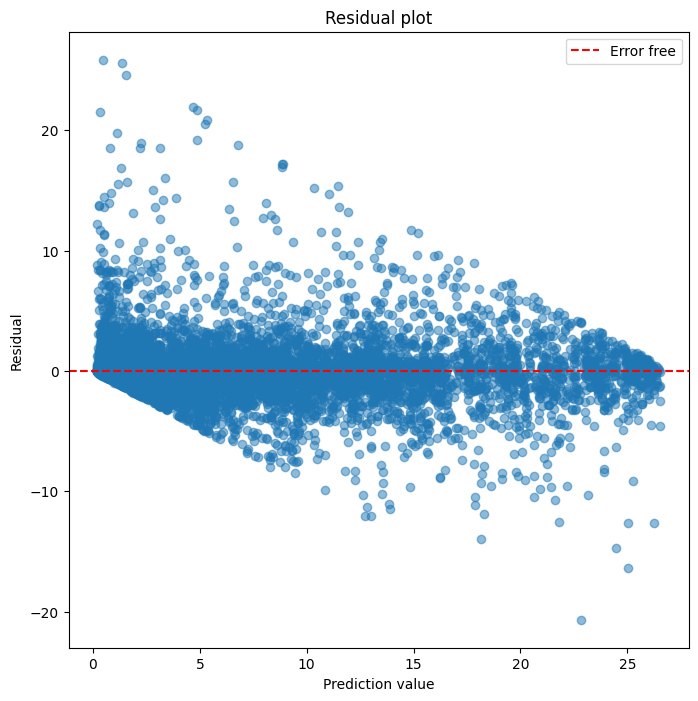


(A) Calibration plot (B) Residual plot

**Figure S7** Throat swab-2nd floor


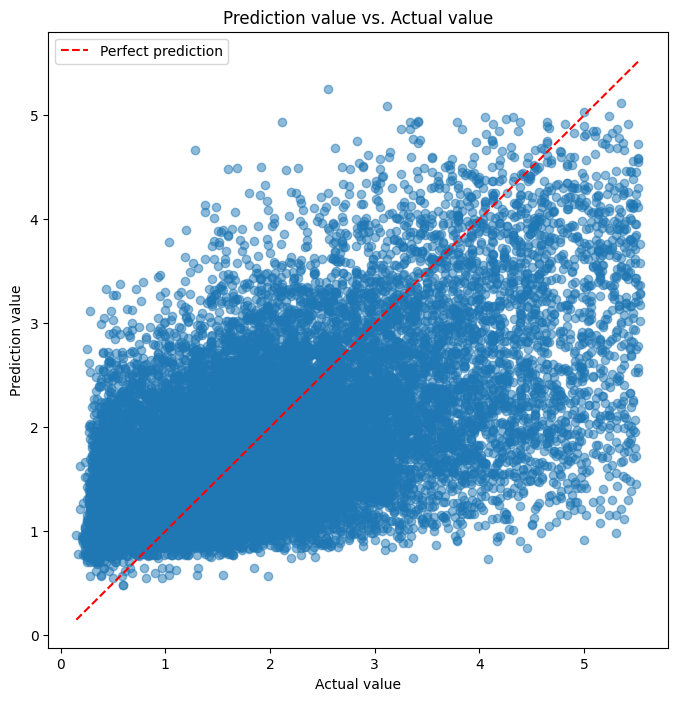

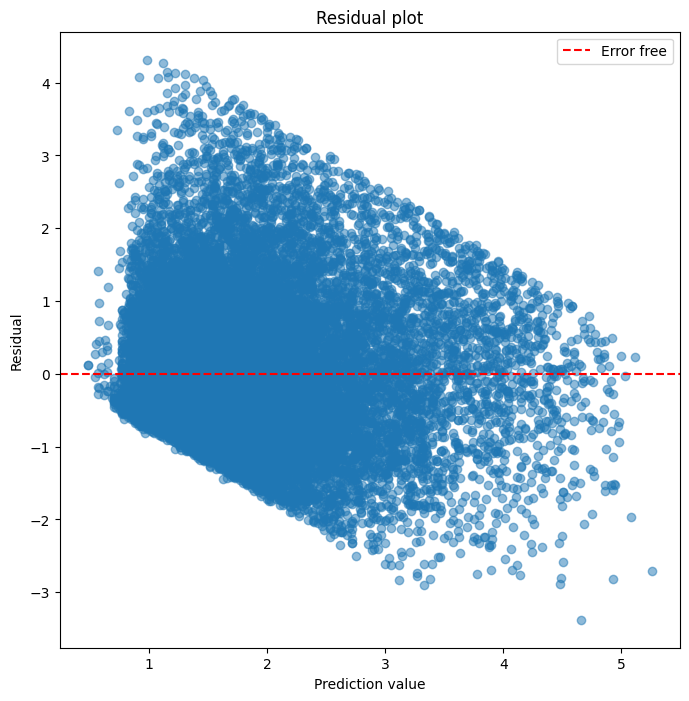


(A) Calibration plot (B) Residual plot

**Figure S8** Blood sampling-ED


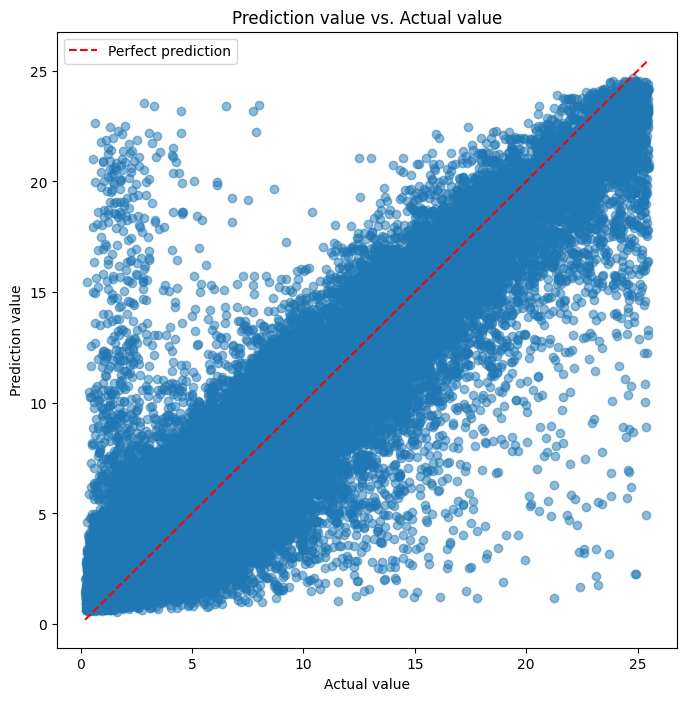

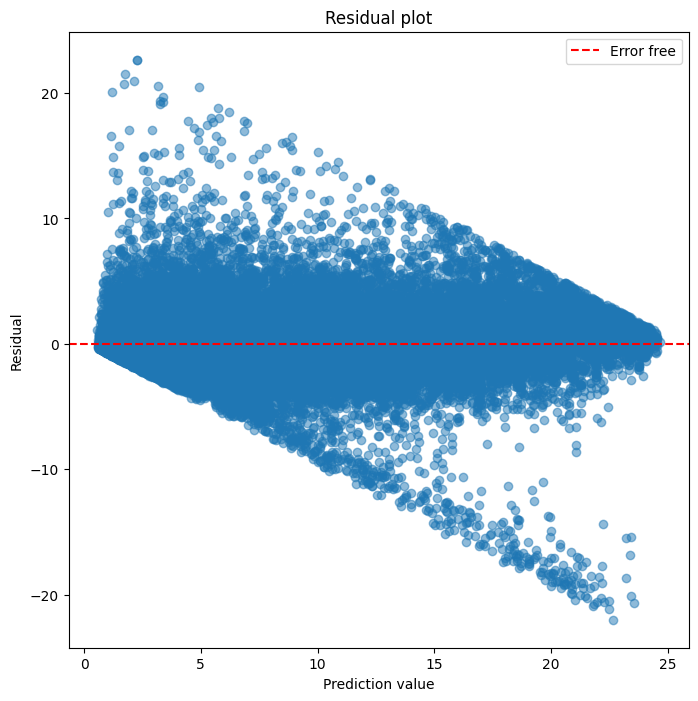


(A) Calibration plot (B) Residual plot

**Figure S9** Blood sampling-OP


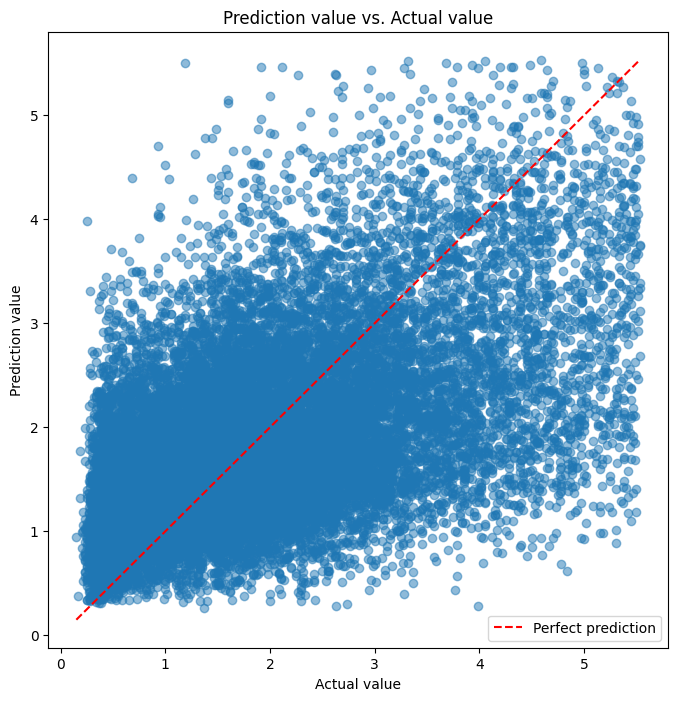

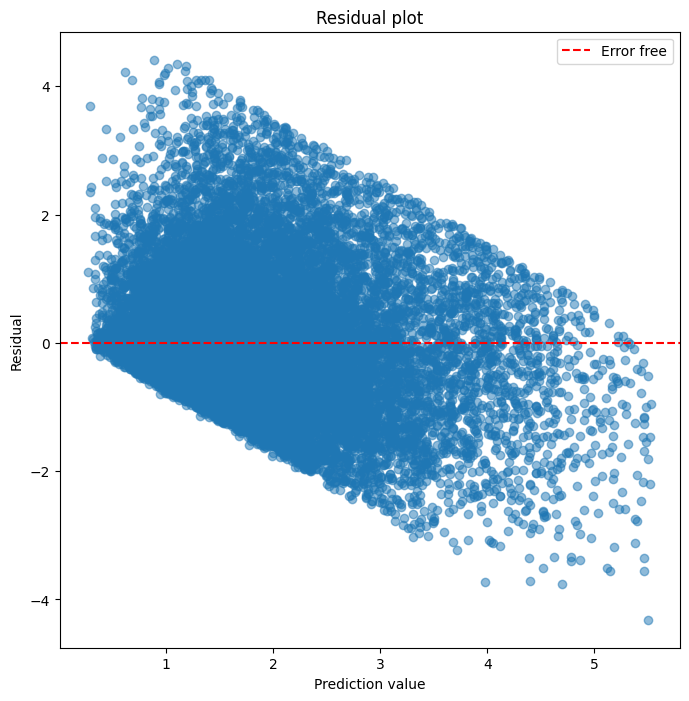


(A) Calibration plot (B) Residual plot

**Figure S10** Laboratory test-Fever


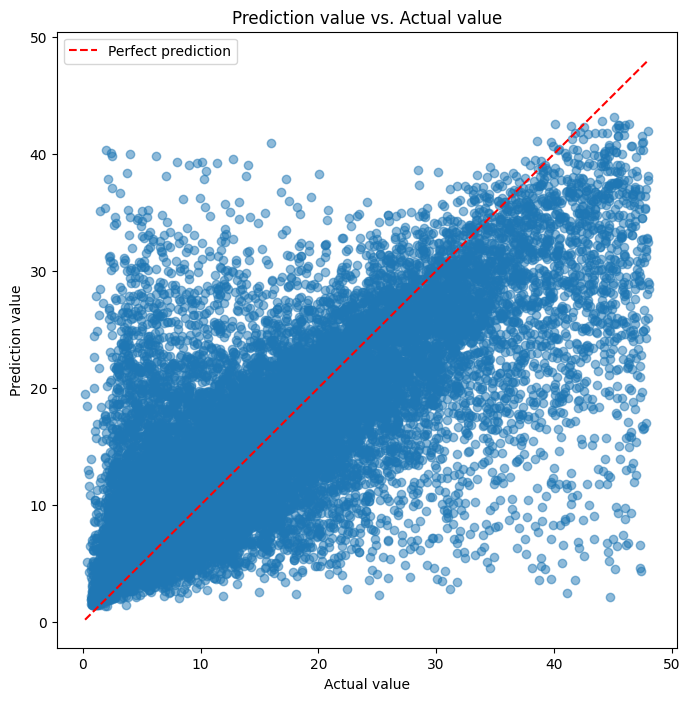

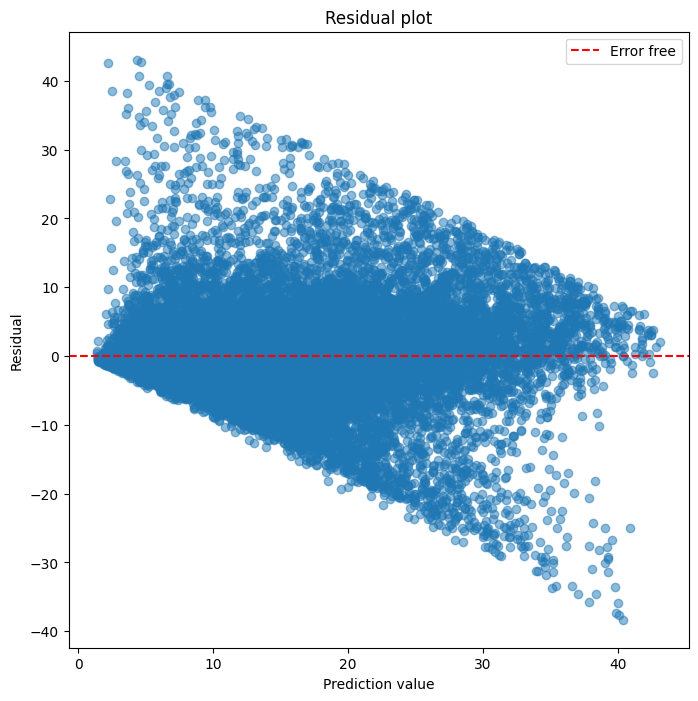


(A) Calibration plot (B) Residual plot

**Figure S11** Ultrasound-ED


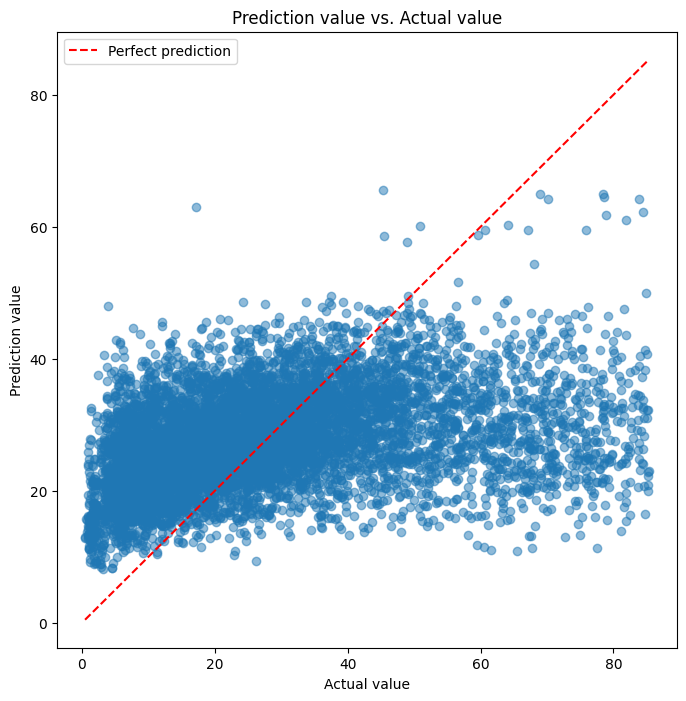

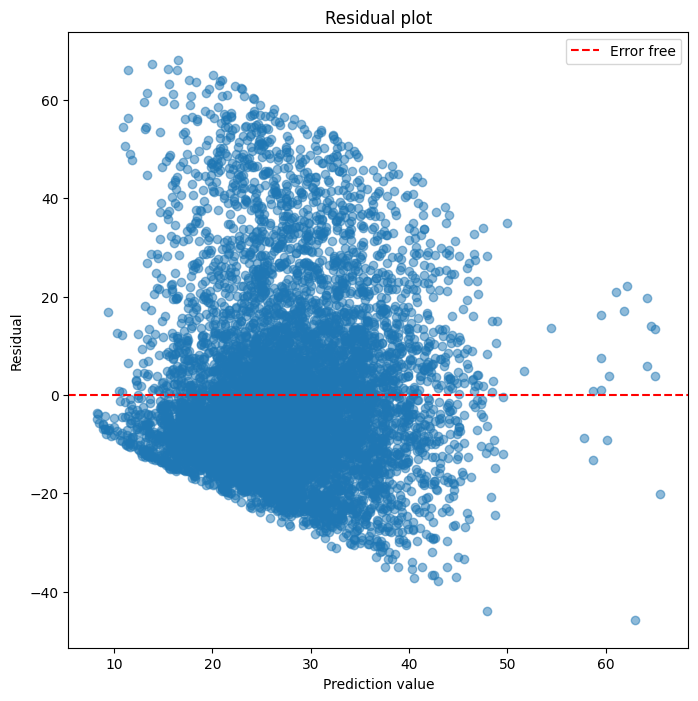


(A) Calibration plot (B) Residual plot

**Figure S12** Ultrasound-OP


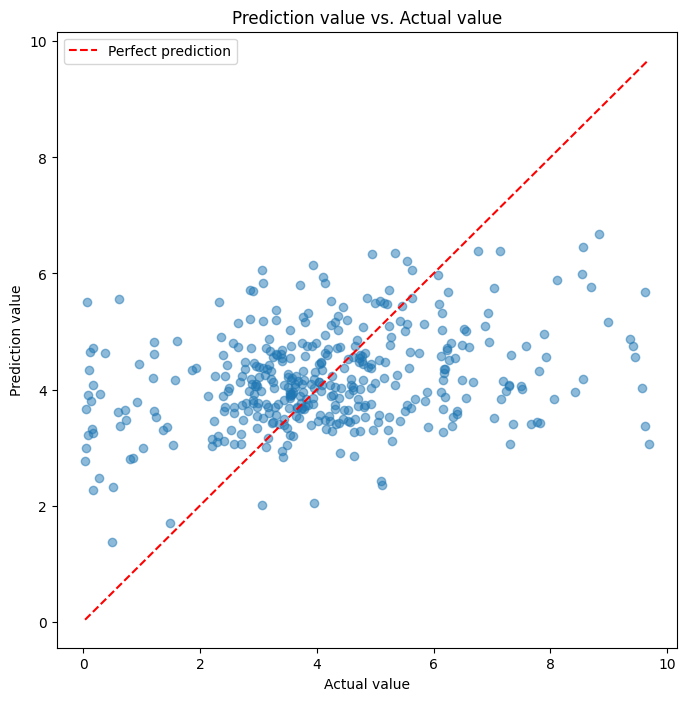

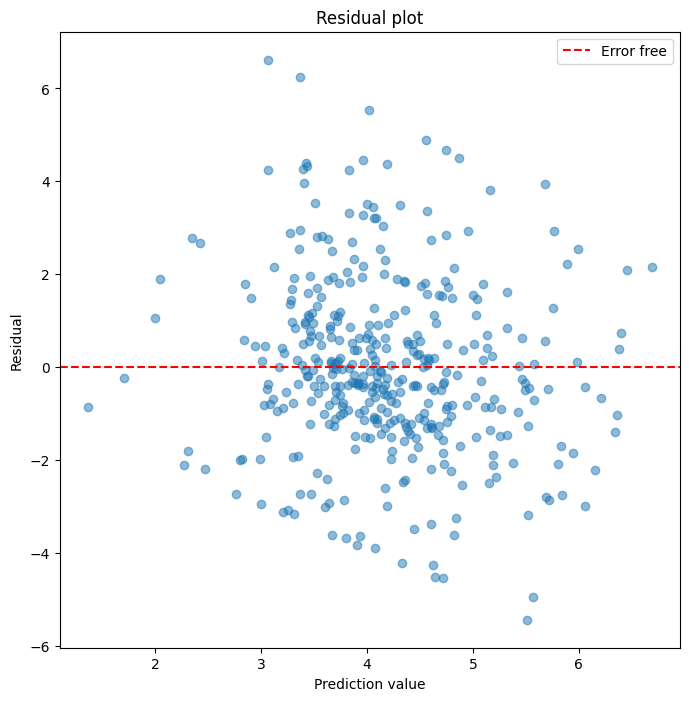


(A) Calibration plot (B) Residual plot

**Figure S13** Echocardiography-ED


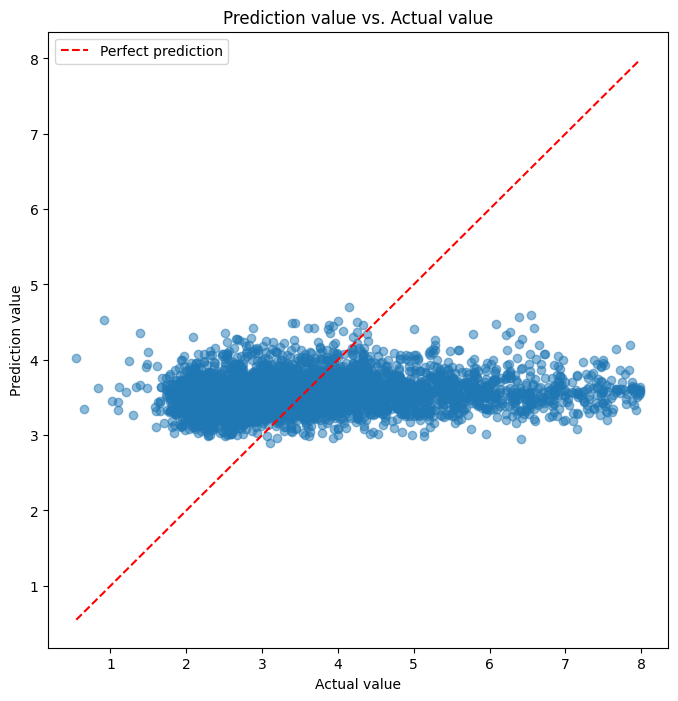

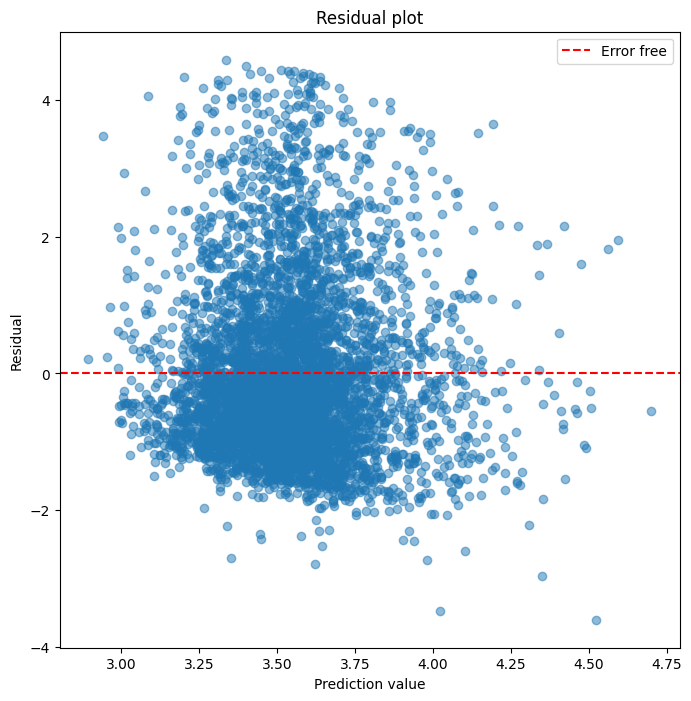


(A) Calibration plot (B) Residual plot

**Figure S14** Echocardiography-OP


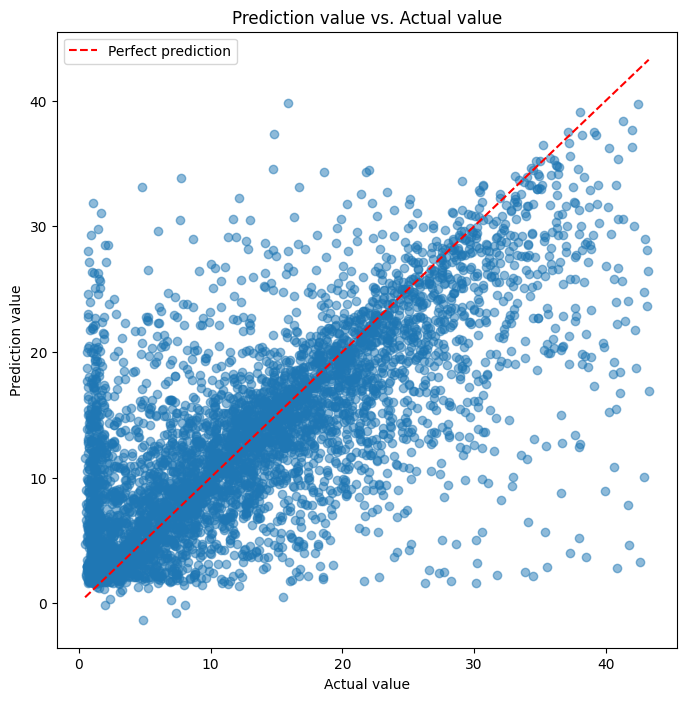

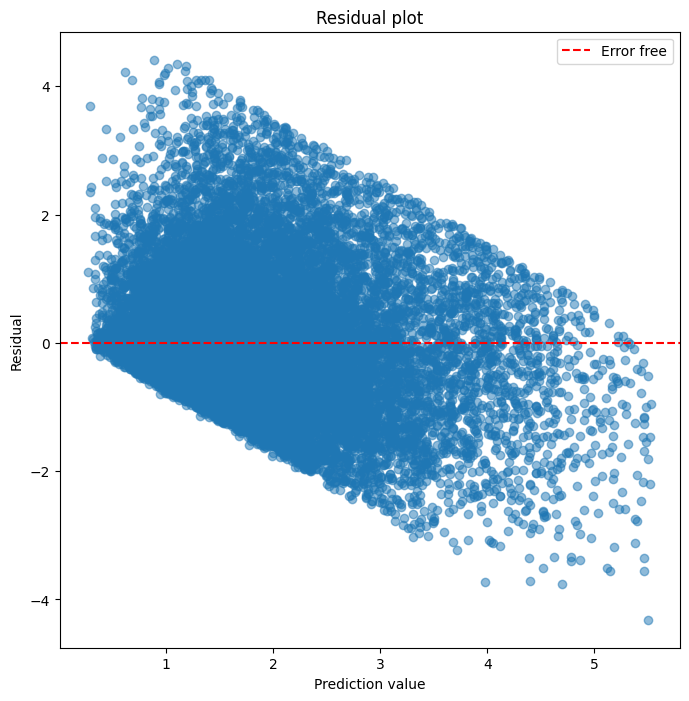


(A) Calibration plot (B) Residual plot

**Figure S15** Laryngoscope


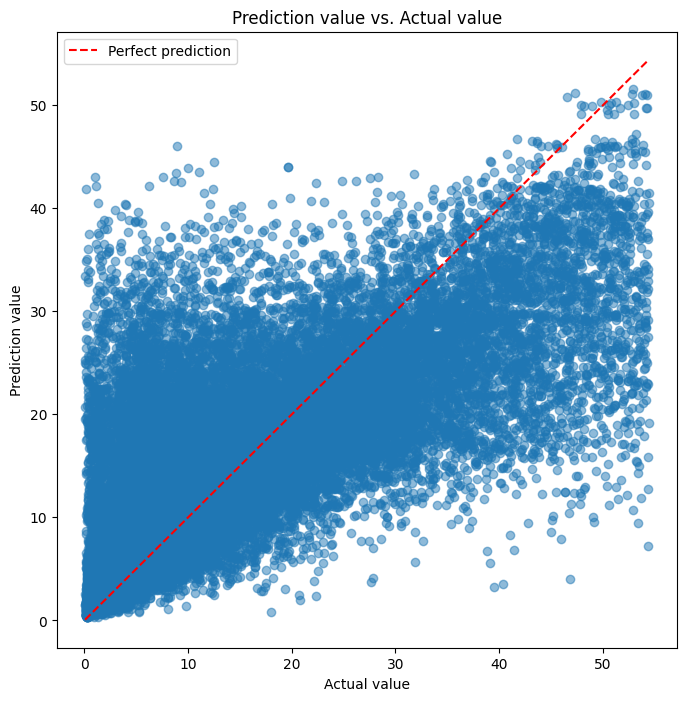

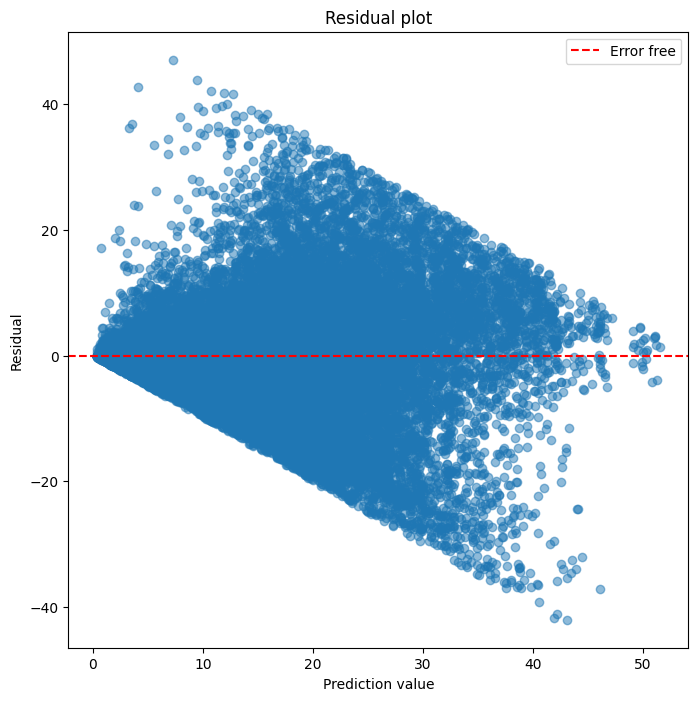


(A) Calibration plot (B) Residual plot

**Figure S16** X ray


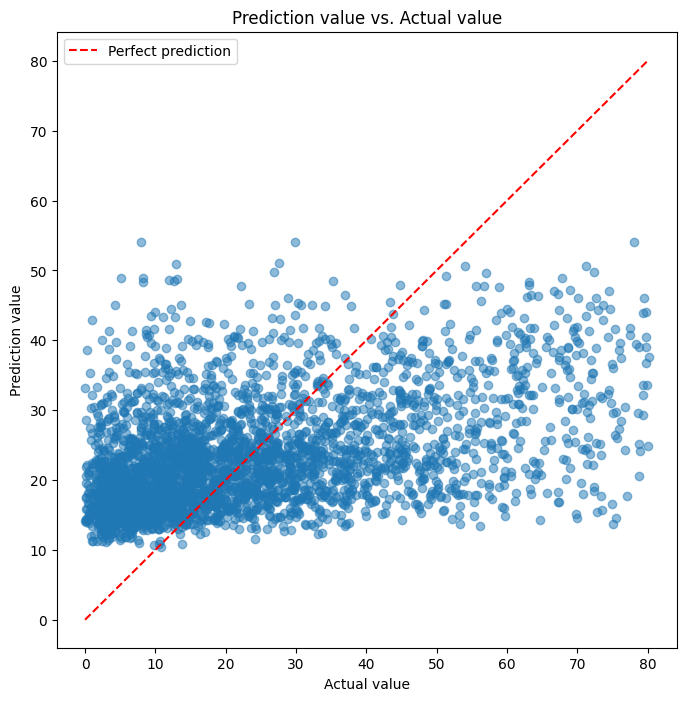

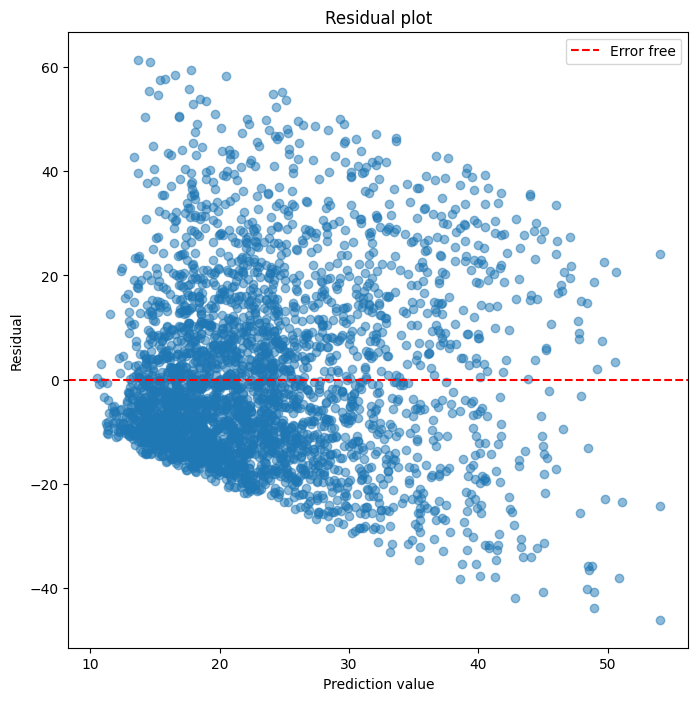


(A) Calibration plot (B) Residual plot

**Figure S17** MRI


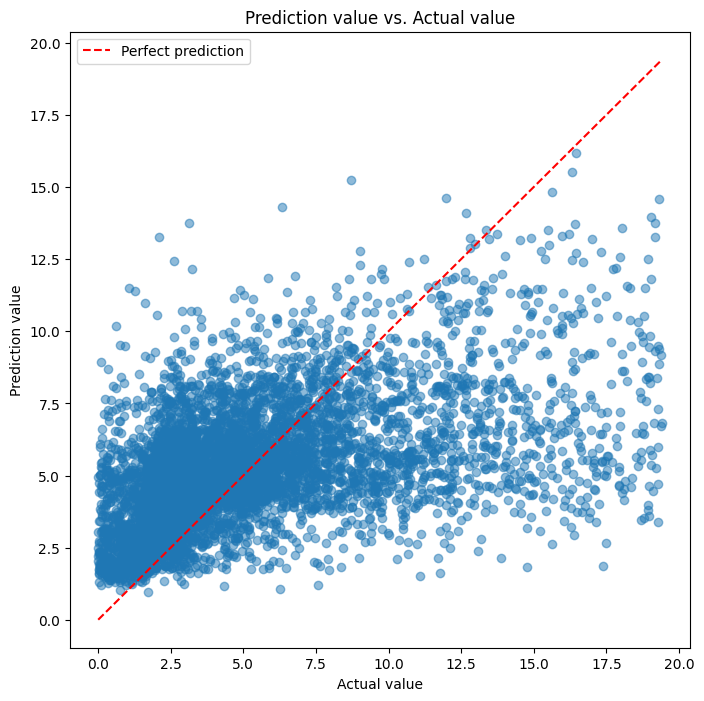

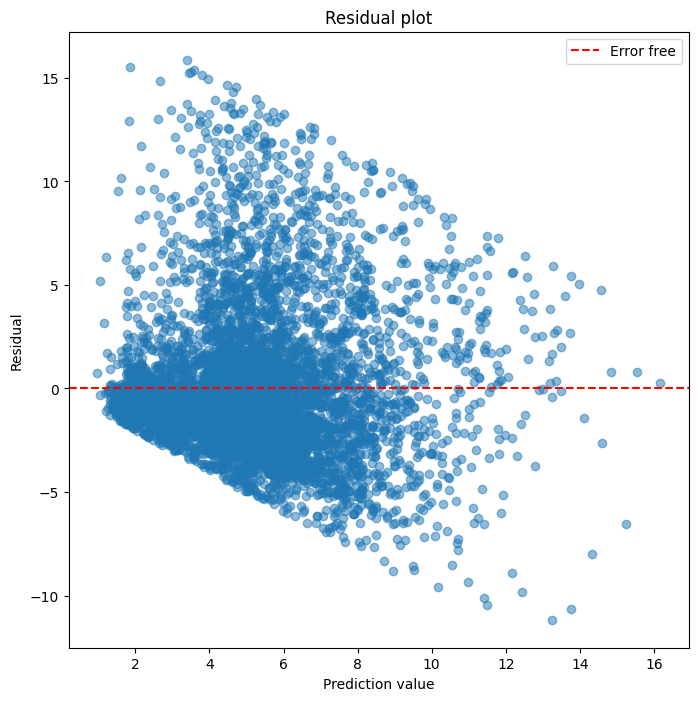


(A) Calibration plot (B) Residual plot

**Figure S18** CT

**Table S3.** The mean SHAP value of each feature in different medical task waiting time prediction

|  | **Month** | **Day** | **Hour** | **the day of week** | **the number of queuing patient** | **Arrival rate** |
| --- | --- | --- | --- | --- | --- | --- |
| Throat swab-1st floor | 0.7068 | 0.2960 | 0.2729 | 0.2482 | 4.3480 | 0.5099 |
| Throat swab-2nd floor | 0.0927 | 0.4828 | 0.3447 | 0.1976 | 4.1969 | 0.4885 |
| Blood sampling-ED | 0.0762 | 0.1084 | 0.1079 | 0.0676 | 0.2608 | 0.1164 |
| Blood sampling-OP | 0.5397 | 0.3554 | 0.2859 | 0.1560 | 3.8139 | 0.3655 |
| Laboratory test-Fever | 0.1468 | 0.2111 | 0.1022 | 0.0987 | 0.8559 | 0.3598 |
| Ultrasound-ED | 0.6636 | 0.5686 | 0.8563 | 0.5047 | 6.6922 | 0.7692 |
| Ultrasound-OP | 0.5993 | 0.9362 | 2.0421 | 1.3370 | 4.3553 | 0.9622 |
| Echocardiography-ED | 0.1496 | 0.3490 | 0.2853 | 0.2273 | 0.0907 | 0.1451 |
| Echocardiography-OP | 0.0472 | 0.0603 | 0.0516 | 0.0764 | 0.0659 | 0.0680 |
| Laryngoscope | 0.7876 | 1.3427 | 0.9882 | 0.8372 | 3.1169 | 1.9548 |
| X ray | 0.6307 | 0.5956 | 1.0510 | 0.4777 | 7.5495 | 1.0537 |
| MRI | 1.0871 | 0.9487 | 1.5861 | 0.9415 | 4.8606 | 1.1836 |
| CT | 0.1951 | 0.2291 | 0.4330 | 0.2061 | 1.2307 | 0.2831 |


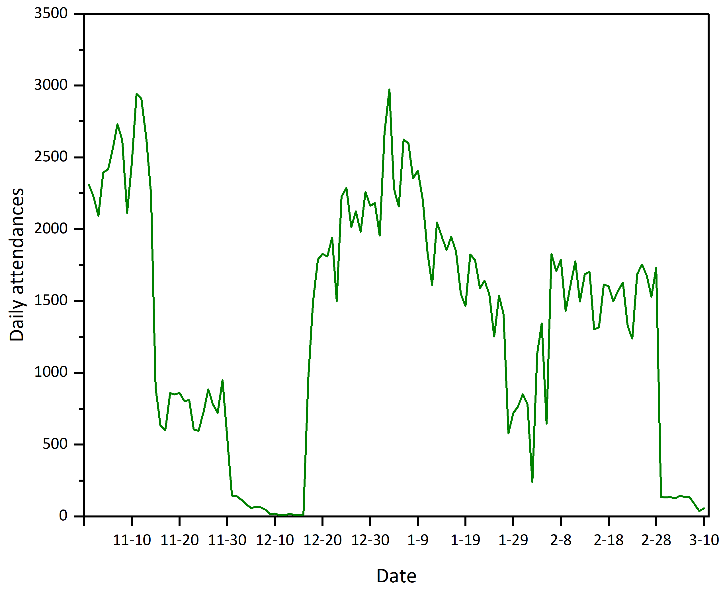


**Figure S19.** Daily laboratory test and radiology examination attendances from Nov.1 2024 to Mar.13 2025


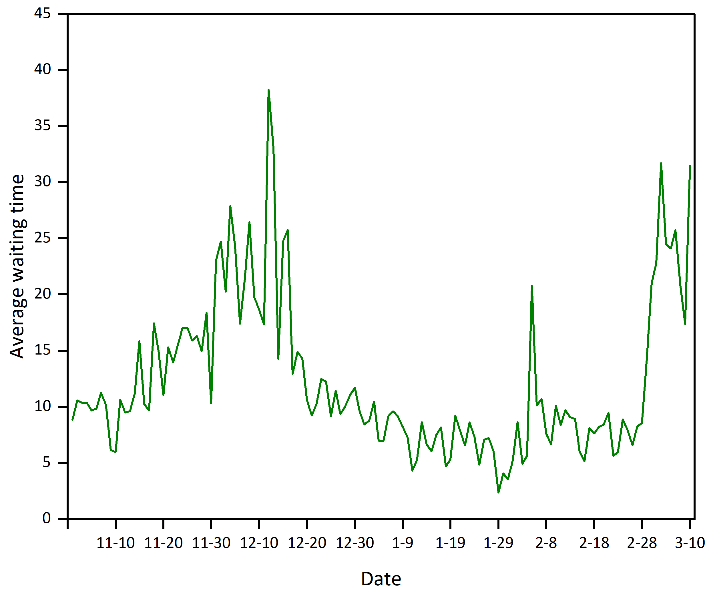


**Figure S20.** Average waiting time from Nov.1 2024 to Mar.13 2025
